# Biosynthesis of very Long‐chain fatty acids is required for *Arabidopsis* auxin‐mediated embryonic and post‐embryonic development

**DOI:** 10.1111/tpj.70396

**Published:** 2025-08-09

**Authors:** David Babić, Rashed Abualia, Lukáš Fiedler, Linlin Qi, Frédérique Tellier, Adrijana Smoljan, Hana Rakusová, Petr Valošek, Huibin Han, Eva Benková, Jean‐Denis Faure, Jiří Friml

**Affiliations:** ^1^ Institute of Science and Technology Austria 3400 Klosterneuburg Austria; ^2^ Université Paris‐Saclay, INRAE, AgroParisTech, Institute Jean‐Pierre Bourgin for Plant Sciences (IJPB) 78000 Versailles France; ^3^ Present address: Biology Department Science and Technology College, Hebron University 40 Hebron, West Bank Palestine; ^4^ Present address: Faculty of Synthetic Biology Shenzhen Institute of Advanced Technology, Chinese Academy of Sciences Shenzhen 518055 China; ^5^ Present address: Institute of Emerging Agricultural Technology Shenzhen University of Advanced Technology, Chinese Academy of Sciences Shenzhen 518055 China; ^6^ Present address: Department of Plant Science McGill University Montreal Quebec Canada; ^7^ Present address: Jiangxi Province Key Laboratory of Vegetable Cultivation and Utilization Jiangxi Agricultural University Nanchang 330045 China

**Keywords:** very long‐chain fatty acids (VLCFAs), sphingolipids, KCR1, meristems, auxin, PINs, *Arabidopsis*, development, endocytosis, trafficking

## Abstract

Very long‐chain fatty acids (VLCFAs), being constituents of different types of lipids, are critical factors in plant development, presumably due to their impact on the endomembrane system. The VLCFAs are synthesized in the endoplasmic reticulum by a heterotetrameric enzymatic complex including β‐ketoacyl CoA reductase 1 (KCR1), whose mutant is lethal. Here, we describe the *ectopic shoot meristems* (*esm*) mutant, a viable *kcr1* allele presumably affecting surface properties of the KCR1 protein. This *kcr1‐2* mutant shows reduced fatty acyl elongation that impacts VLCFAs. The *kcr1‐2* plants show severe defects during different stages of development, which all correlate with defects in polar localization and subcellular trafficking of PIN auxin transporters and resulting asymmetric auxin distribution. Detailed analysis of KCR1 expression and patterning defects in *kcr1‐2* suggests that KCR1 plays a role in delineating boundaries around meristematic and specialized differentiating tissues, including root and shoot meristems, initiating lateral roots, lateral root primordia, and trichomes. In these contexts, KCR1‐produced VLCFAs may act in a non‐cell‐autonomous manner. Viable *kcr1‐2* represents a useful tool to study VLCFA roles in plant development and highlights VLCFAs as critical developmental factors at the interface of cell polarity and tissue development.

## Significance Statement

The crucial link between auxin‐mediated development and lipid biology in *Arabidopsis* has been long established, but insufficiently studied due to embryo lethality of mutants involved in these biological processes. In this study, authors have isolated a viable mutant of *KCR1*, a gene involved in fatty acid biosynthesis, and have shown a connection between lipid biosynthesis, auxin, and plant development. The mutant shows defects in embryogenesis and organogenesis, involving a novel phenotype – extranumerary vegetative meristems.

## INTRODUCTION

The patterning of plant tissues and organs is based on spatiotemporally coordinated cell divisions and differentiation, both of which are controlled by a number of molecular players (Meyerowitz, 1997). Although the *status quo* of meristematic cell identity and differentiation of plant tissues relies on intrinsic genetic and positional information, the phytohormone auxin is known to serve as an important signal for dynamic changes in developmental programs (Friml, 2022; Vanneste et al., 2025). Auxin plays a key role in many developmental contexts including (i) shoot apical meristem (SAM) and lateral root organogenesis (Benková et al., 2003; Heisler et al., 2005), (ii) embryogenesis (Friml et al., 2003; Robert et al., 2018), (iii) primary root patterning (Billou et al., 2005; Ding & Friml, 2010; Rahman et al., 2007), and (iv) leaf morphogenesis and venation (Hajný et al., 2022; Kierzkowski et al., 2019; Scarpella et al., 2010; Xiong & Jiao, 2019). The establishment and maintenance of local auxin maxima and gradients within plant tissues form the basis for these developmental phenomena (Benková et al., 2009; Vanneste & Friml, 2009). The directional intercellular auxin fluxes, which to a large extent underlie the auxin distribution, depend primarily on the asymmetric deposition of PIN auxin transporters at plasma membranes (Adamowski & Friml, 2015; Wisniewska et al., 2006). This dynamic cellular PIN polarity is linked to constitutive PIN endomembrane trafficking (Adamowski & Friml, 2015; Narasimhan et al., 2021). Recent research suggests that PIN sorting and vesicle trafficking depend on sphingolipid membrane composition, among other factors (Markham et al., 2011). Sphingolipids are a diverse class of lipids derived from amino alcohol long‐chain base (LCB) N‐acylated with fatty acids (FA) to form ceramides (Cers) or hydroxyceramides (hCers). The attachments of a polar head group to the LCB moiety of Cers and hCers produce complex sphingolipids such as glucosylceramides (GlcCers) and glycosyl inositol phosphoceramides (GIPCs) (Shayman, 2000). Different types of plant cell membranes are characterized by distinct sphingolipid species or their ratios (Liu et al., 2021), potentially defining endomembrane regions for polar protein targeting (Markham et al., 2011). The major fatty acid constituents of sphingolipids include VLCFAs, which are defined as FA with acyl chains of at least 20 carbon atoms in length (Haslam & Kunst, 2013). The biosynthesis of VLCFAs occurs through the action of four enzymes bound to the endoplasmic reticulum (ER) that form a heterotetrameric complex—the fatty acid elongase complex. Mutants of different components of this complex, or of associated cytoplasmic enzymes such as *pasticcino3*/*gurke*, have a reduced VLCFA content and show severe defects in tissue pattern formation (Faure et al., 1998) or are embryo lethal like the 3‐hydroxy‐acyl‐CoA dehydratase PASTICCINO2 (Bach et al., 2008). Another component of the plant fatty acid elongase complex, β‐Ketoacyl‐Coenzyme A Reductase1 (KCR1), was first identified by complementing a yeast mutant with the plant KCR1 protein, and while the RNAi knock‐down *kcr1* alleles have defective plant morphology, the T‐DNA insertion mutant (*kcr1‐1*) is embryo lethal (Beaudoin et al., 2009). Altogether, these findings showed the essential role VLCFAs have in plant development. However, detailed insights into their developmental roles and underlying cellular mechanisms remain limited.

Here, we describe a viable *kcr1‐2* allele, displaying severe patterning defects and ectopic cell proliferation, including extranumerary vegetative SAMs and other auxin‐related developmental phenotypes, which provide new insights into the roles of VLCFAs in PIN‐dependent auxin transport and defining boundaries for cell division and differentiation.

## RESULTS

### Identification of *ectopic shoot meristems* (*esm*) mutant with severe apical patterning defects

**Figure 1:**
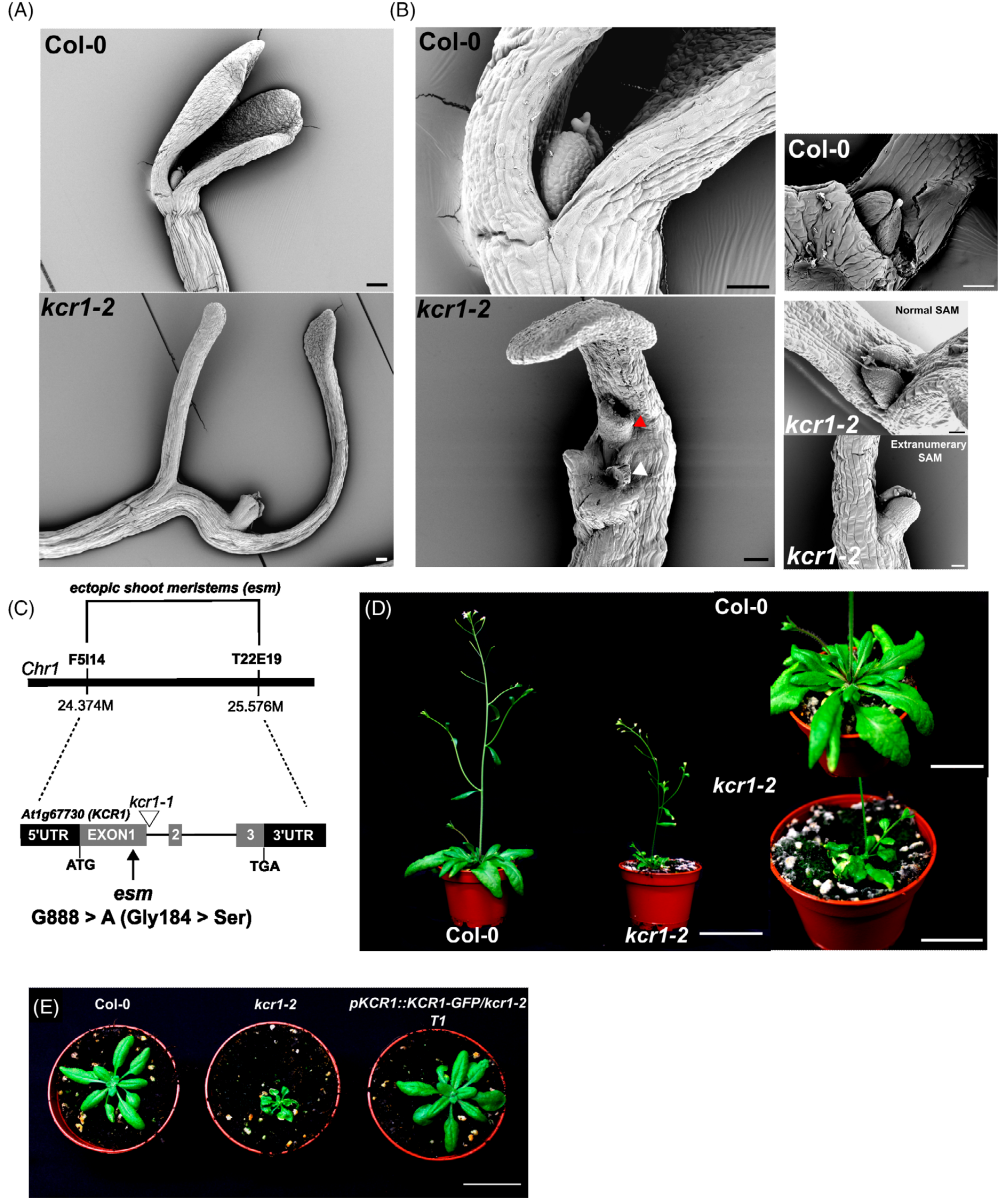
Cotyledon phenotypes and gene mapping of *esm* (*kcr1‐2*). (A) Scanning electron microscopy (SEM) images of cotyledons and vegetative apical meristems of 4‐day‐old dark‐grown seedlings. (B) SEM images of wild‐type vegetative apical meristem (upper panels); apical (white arrowhead) and extranumerary (red arrowhead) vegetative meristems of *esm* (*kcr1‐2*) plants (lower panels). (C) Schematic of the *esm* mutation mapping. The *KCR1* gene (At1g67730) is found on the first chromosome and has three exons. The *esm* mutation (Glycine 184 mutated to Serine) maps to the first exon. Previously published *kcr1‐1* T‐DNA mutation position is labeled by an inverted triangle. (D) Phenotypes of adult *kcr1‐2* plants. Left panel – flowering plants; Right panel – adult rosette. (E) Complementation of *kcr1‐2* phenotype by pKCR1::KCR1‐GFP construct. Scale bars: A – 100 μm, B – 50 μm; D – Left image 5 cm; right images – 3 cm; E – 3 cm.

With a primary interest in identifying genes important for hypocotyl gravitropism, we performed an ethyl methanesulfonate (EMS) screen on *pPIN3::PIN3‐GFP* plants and focused on mutants exhibiting impaired bending upon gravistimulation, as described previously (Rakusová et al., 2019). Unexpectedly, beyond typical agravitropic and hyperbending mutants, we recovered a mutant with severe defects in apical patterning. In contrast to etiolated wild‐type (WT) cotyledons that form an apical hook, cotyledons of this mutant were completely malformed and often lacked leaf‐like structures or exhibited a stumped, stem‐like outgrowth (Figure 1A). Another striking phenotype was the appearance of extranumerary SAMs (Figure 1B). Based on these unusual defects, we named the mutant *ectopic shoot meristems* (*esm*).

### *esm* carries a mutation in the *KCR1* gene

To map the *esm* mutation, we performed a cross with Col‐0 WT plants and observed that the *esm* mutation was recessive (Figure 1A). Next, we used primary chromosomal mapping and determined the approximate position of *esm* between two genetic markers on chromosome 1 – F5I14 and T22E19. Subsequent examination of this region by next generation sequencing revealed three lesions (Table S1), including one in the *KCR1* gene (At1g67730). Given that the phenotypic defects of *esm* plants partially overlapped with those of RNAi lines targeting the *KCR1* (Beaudoin et al., 2009), we further focused on the *KCR1* lesion and performed an allelic test. A cross with the heterozygote of the published embryo‐lethal *kcr1‐1* mutant (Beaudoin et al., 2009) did not rescue the recessive *esm* mutation, confirming that *esm* is indeed allelic to *kcr1‐1* (Figure S1). This notion was further confirmed by transformation of the *pKCR1::KCR1‐GFP* fusion construct into *kcr1‐2* plants, which led to a full complementation of the phenotype defects (Figure 1E). This led us to rename *esm* to *kcr1‐2*. We conclude that the *kcr1‐2* (*esm*) phenotype is a consequence of a missense point mutation (DNA: G888A; protein: Gly184Ser) in the first exon of the *KCR1* gene (Figure 1C), whose protein product is crucial for a rate‐limiting step in VLCFA biosynthesis (Roudier et al., 2010).

### *kcr1‐2 (esm)* causes reduced fatty acyl elongation in seedlings

**Figure 2:**
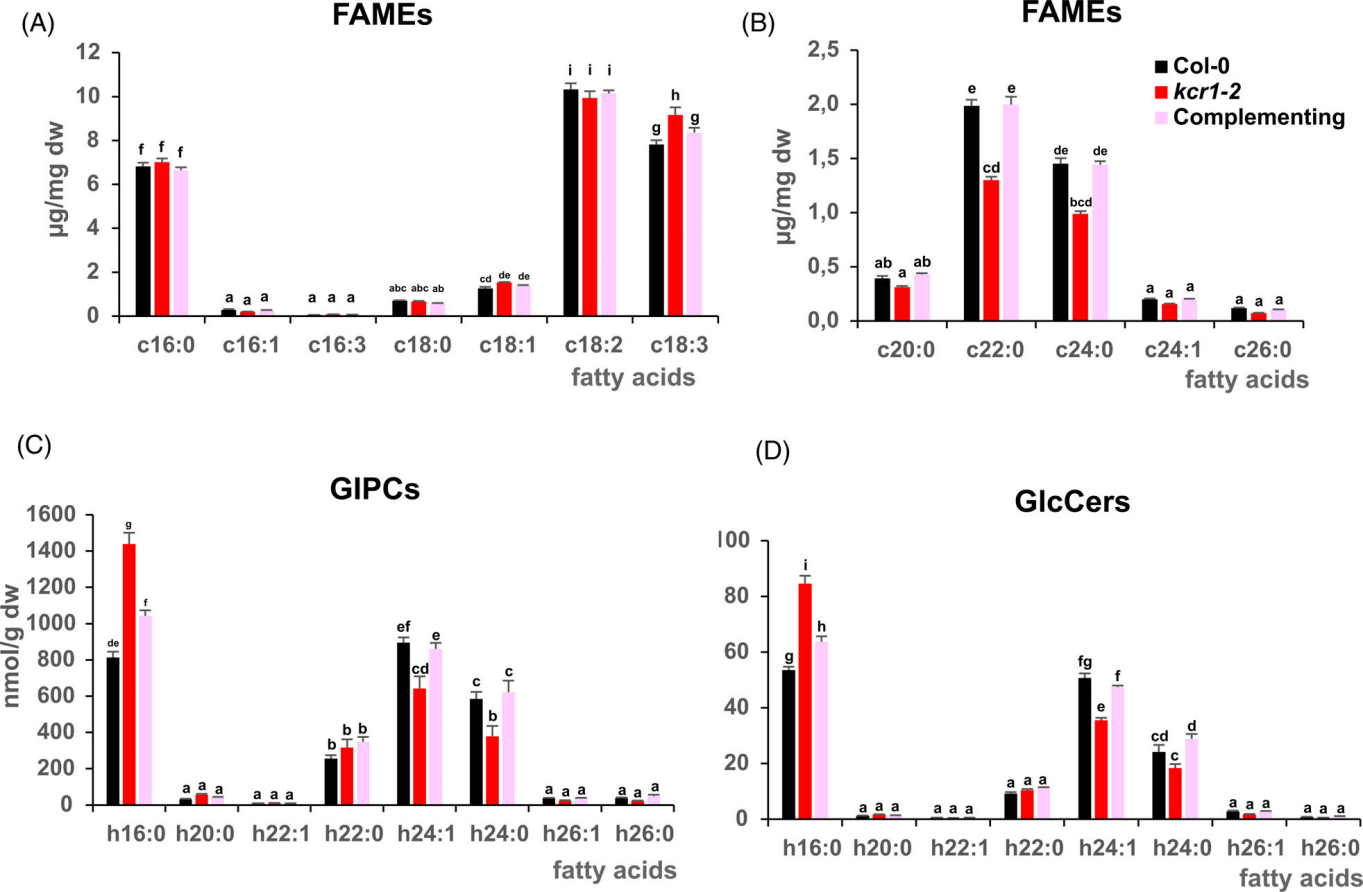
Reduced VLCFA content in *kcr1‐2* seedling roots. (A, B) Fatty acyl methyl esters (FAMEs) profile in *kcr1‐2* mutant roots compared with wild‐type and complemented line. Total fatty acid levels in roots of *kcr1‐2* mutant and complementing line compared with Col‐0 (amounts expressed in μg/mg dry weight). Fatty acid values are the average of four samples ± SD. (C, D) Glycosyl inositol phosphoceramides (GIPC) (C) and glucosylceramides (GlcCer) (D) profiles in roots of *kcr1‐2* mutant compared with wild‐type and complemented line. GIPC and GlcCer amounts are shown according to their fatty acid length. For each hydroxylated (h) fatty acid, the values correspond to the sum of the four LCBs isoforms. Sphingolipid values are the average of three samples ± SD. Two‐way ANOVA with fatty acid:genotype interaction with a *post hoc* for the different lipid classes using genotype and FA types as first and second grouping variables was used. Color codes for genotypes are the same for all panels and shown in panel B.

To evaluate whether *kcr1‐2* mutation modified KCR1 activity and fatty acyl elongation, we analyzed fatty acid methyl esters (FAMEs) profiles of roots and shoots of *kcr1‐2* seedlings compared with WT (Figure 2A,B; Figure S2A,B). In roots where VLCFAs are the most abundant, *kcr1‐2* showed a reduction of the most abundant c22:0 and c24:0 FA with a decrease corresponding to 34 and 32% of WT values (Figure 2B). The accumulation of the less abundant VLCFAs c20:0, c24:1, and c26:0 was also reduced (respectively 20, 22, and 30% reduction compared with WT). A similar reduction was also observed in shoots for the most abundant c24:0 and c26:0 with a decrease corresponding respectively to 30% and 39% of WT values (Figure S2B). VLCFA reduction in *kcr1‐2* was completely restored in *pKCR1::KCR1‐GFP*/*kcr1‐2* complementing line, demonstrating that the Gly184Ser mutation in KCR1 was leading to significant reduction of fatty acyl elongation in seedlings.

It was previously shown that VLCFAs are major constituents of sphingolipids. We therefore measured sphingolipid content in *kcr1‐2* mutant seedling roots and shoots. The most abundant sphingolipids were the membrane GIPC and GlCcers with the hydroxylated fatty acyl chain h16:0, h22:0, h24:0, and h24:1 (Figure 2C,D; Figure S2E,F). As expected, the VLCFA GIPC and GlCcers decreased in *kcr1‐2* (−28% to 43% reduction in roots; −2% to 24% in shoots) while the h16:0 isoforms increased (+58 to 109%). There was one exception, with h22‐GIPC and ‐Glucers in roots increased in *kcr1‐2* (Figure 2C,D). Similar findings were observed in *pas3* elongation mutants (Roudier et al., 2010). The less complex sphingolipid Cer and hCers showed a similar pattern in both roots and shoots (Figure S2C,D,G,H). Like for the FAMEs, these sphingolipid changes were restored to WT levels in the complemented lines, indicating that kcr1‐2 mutation was directly impacting fatty acyl chain homeostasis of sphingolipids.

### *kcr1‐2* (*esm*) phenotype analysis

We first investigated the phenotypes of dark‐grown *kcr1‐2* seedlings. These manifested as hypocotyl and cotyledon defects (Figure 1A). In line with our original screen for hypocotyl gravitropism defects, the etiolated *kcr1‐2* hypocotyls show bending defects, are significantly shorter than the WT seedlings, and never develop an apical hook (Figure S6A,B). The most frequently observed cotyledon defect (51% of all seedlings) was that one cotyledon developed almost normally, while the second was smaller and retarded (Figure S1). In about one‐third of the *kcr1‐2* seedlings (35%), one cotyledon was abnormally elongated, while the other cotyledon did not form at all. In the extreme cases (14%), cotyledons completely failed to develop, leading to a stump‐like apex (Figure S1). Similar defects have been described for other mutants deficient in VLCFA biosynthetic enzymes—*pas1*, *pas2*, and *pas* (Faure et al., 1998) *3*. The *kcr1‐2* mutant exhibited an additional extreme phenotype: the appearance of extranumerary SAMs. Besides the usual SAM located between cotyledons or cotyledon primordia (Figure 1B), *kcr1‐2* plants showed additional SAMs developing in abnormal positions along the stem, for example, on the hypocotyl or in the vicinity of the normal SAM (Figure 1B). The SAM structure itself was normal, with trichome‐covered leaf primordia growing in a usual WT pattern (Figure 1A,B). However, we occasionally observed seemingly overproliferating SAMs (Figure S1). To the best of our knowledge, the striking'extranumerary SAM' phenotype has not been described previously for any of the VLCFA‐related mutants or for any other *Arabidopsis* mutants. However (Faure et al., 1998) describe clusters of dense, non‐vacuolated cells in cotyledons and leaves of *pas2* mutants reminiscent of meristematic cells, which could be the structures we ourselves observed. This observation could link the apical meristematic identity with the action of VLCFAs.

Light‐grown *kcr1‐2* seedlings also showed apical patterning defects, albeit to a lesser extent (Figure S1). The most common phenotype was that only one cotyledon formed normally (44% of all seedlings). These would often appear as small, chlorotic, corn seed‐like seedlings (Figure S1). Adult *kcr1‐2* plants were significantly shorter than WT (Figure 1D) and exhibited folded and curled rosette leaves (Figure 1D). Furthermore, the mutant plants were hypersensitive to excess water, as evidenced by the fact that they had to be irrigated suboptimally to achieve successful propagation in soil. This phenotype is in contrast to what has been previously described for *kcr1* RNAi mutants (Beaudoin et al., 2009).

In general, the *kcr1‐2* plants showed many phenotypic features typical of plants defective in VLCFA biosynthesis (Beaudoin et al., 2009; Shang et al., 2016) with the exception of ectopic SAMs. Nonetheless, the low‐penetrance “overproliferating SAM” phenotype has been described previously in *pas2* mutants (Nobusawa et al., 2013) and has been associated with VLCFAs, which are thought to confine the apical meristematic zone. Furthermore, PUCHI, a transcription factor known to regulate the expression of molecular components for VLCFA biosynthesis (Trinh et al., 2019), has been likewise implicated in regulating meristem‐related gene expression and therefore in inhibiting the ectopic establishment of meristematic cell identities (Bellande et al., 2022). Whether the extranumerary SAMs of *kcr1‐2* are merely an extreme manifestation of overproliferation or whether they represent a novel defect reflecting the unique function of KCR1 remains unclear.

### Potential structural effects of the *kcr1‐2* mutation

To estimate the molecular effect of the *kcr1‐2* missense mutation on KCR1 protein, we modeled the structure of KCR1^WT^ (Uniprot ID: Q8L9C4) using AlphaFold3 (AF3) (Abramson et al., 2024). The results showed very high model confidence (Figure S2A). As expected from a member of the widespread short‐chain dehydrogenase/reductase (SDR) superfamily (Lukacik et al., 2006), KCR1 encompasses a “Rossmann fold” (an αβα sandwich) which binds NADPH during VLCFA synthesis (Figure 2A). Myristic acid molecules were included as ligands in the modeling, and according to expectation, showed attraction toward a nonpolar α‐helix annotated as a transmembrane domain in public databases (Figure 2A,C–E).

**Figure 3:**
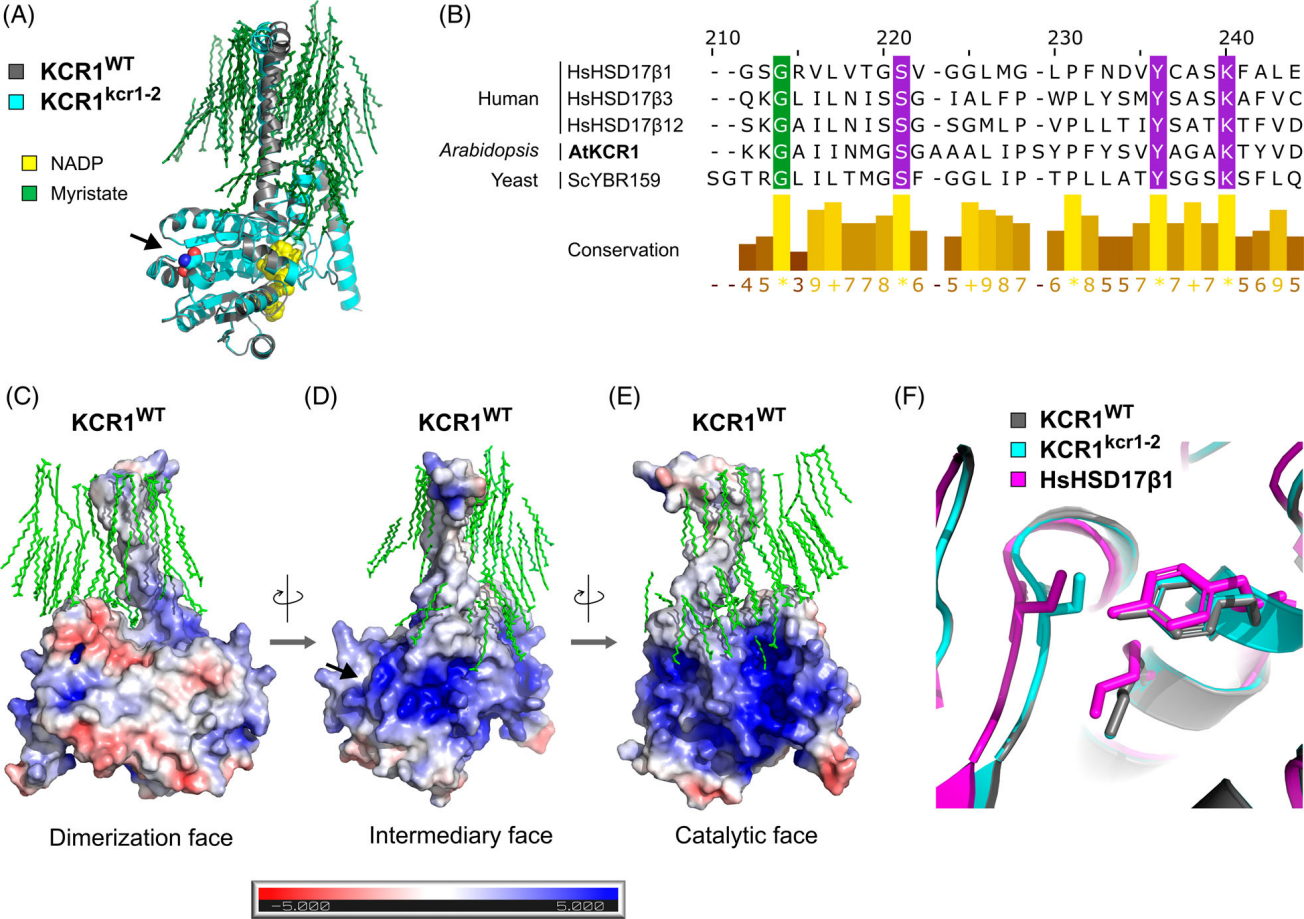
Inferring the structural effect of the *kcr1‐2* missense mutation on the KCR1 protein. (A) AlphaFold3 model of the KCR1^kcr1–2^ mutant protein (cyan) superimposed with that of wild‐type KCR1 (Uniprot ID: Q8L9C4, gray). Black arrow highlights the G184S *kcr1‐2* missense mutation. Coloring is arbitrary and does not reflect AF3 model confidence. (B) The amino acid targeted by *kcr1‐2* (green highlight) is conserved between *Arabidopsis*, humans, and yeast. Depicted are the human ortholog of KCR1 (17β‐hydroxysteroid dehydrogenase 12, Uniprot ID: Q53GQ0), two other human 17β‐hydroxysteroid dehydrogenases (Uniprot IDs: P14061 and P3705B; also see PDB ID: 1BHS), and the yeast ortholog of KCR1 (Uniprot ID: P38286). A conserved catalytic motif typical of the SDR superfamily is highlighted in magenta. (C–E) Maps of electrostatic surface potential for AlphaFold3 protein models of KCR1^WT^ showing presumed dimerization, intermediary, and catalytic faces of the protein. The units are k_B_T/ec ([Boltzmann constant × temperature] /the charge of an electron) and come from the solution of the Poisson–Boltzmann equation. Black arrow highlights the site of the G184S *kcr1‐2* missense mutation. (F) Analysis of the KCR1 active site. Comparison of catalytic motifs for KCR1^WT^ (AlphaFold2 structure, gray), KCR1^kcr1–2^ (AlphaFold2 structure, cyan), and human 17β‐hydroxysteroid dehydrogenase 1 (crystal structure, PDB ID: 1BHS). The Ser‐Tyr‐Lys catalytic triad is depicted as a stick model.

Multiple sequence alignment demonstrated that KCR1 possesses an archetypal catalytic triad typical of the SDR superfamily (Beaudoin et al., 2009; Lukacik et al., 2006). Indeed, the glycine targeted by *kcr1‐2* maps only six amino acids away from the beginning of the catalytic triad, and remarkably, is conserved between *Arabidopsis*, human, and yeast (Figure 3B). We next used an available crystal structure of the human 17β‐hydroxysteroid dehydrogenase type I (the closest homolog of KCR1, for which the structure is known (Ghosh et al., 1995)) to examine the KCR1 catalytic motif. The active site of KCR1^WT^ showed good agreement with the published crystal structure, supporting the validity of AF3 models (Figure 2F).

Next, we mapped the electrostatic surface potential for the KCR1^WT^ AF3 structural prediction. This allowed us to define three faces of the protein. First, we observed a largely nonpolar platform (Figure 2C). Crucially, oligomerization is a common property among the SDR superfamily members (Lukacik et al., 2006), and the nonpolar platform correlates with a high‐confidence KCR1^WT^ dimerization interface predicted by AF3 (Figure S2C,D,E). The physicochemical properties of the protein further allowed us to predict an intermediary face of the protein (Figure 2D) and a presumed catalytic face (Figure 2E), which shows net positive charge and contains an NADPH binding cleft. This is in agreement with published annotations of NADPH binding residues in KCR1 (Beaudoin et al., 2009; Ghosh et al., 1995).

Having characterized the KCR1^WT^ AF3 prediction, we also modeled the KCR1^kcr1–2^ protein. Although AF3 cannot predict the effect of missense mutations on protein structure (Pak et al., 2023), we reasoned that AF3 predictions might still guide testable hypotheses about the structural effects of *kcr1‐2*. The mutation is found close to the protein surface at the base of a β‐strand within the Rossmann fold of KCR1 (Figure 2A and S2A). This site resides at the intermediary face of KCR1 (Figure 2D), suggesting that *kcr1‐2* might not interfere with NADPH binding or KCR1 dimerization (Figure 2A; Figure S2A–E). The mutation replaces a buried hydrogen side chain (glycine) with a bulky hydroxymethyl group (serine). To accommodate this change, the nearby peptide backbone probably becomes slightly distorted, which is consistent with KCR1^kcr1–2^ but not KCR1^WT^ AF3 predictions showing low confidence in regions surrounding the lesion (Figure S2B,E). This presumably has a knock‐on effect on a nearby Arg49, which gets shifted and bulged out of the protein in all predicted structures (Figure S2A,B). These changes seem to have the potential to alter the electrostatic surface potential distribution, especially around the *kcr1‐2* mutation in both KCR1^kcr1–2^ monomers and dimers (Figure S2A–E).

We reasoned that alterations of surface charge might reprogram the protein–protein interactions of KCR1 and used AF3 to predict potential interfaces between KCR1^WT^ and its known interactors from the fatty acid elongase complex (Kim et al., 2022): KCS9, ECR, and PAS2. These models showed very low overall support for the interactions with KCR1, even when we screened various stoichiometries, cofactors, and membrane‐like ligands. In a small number of cases, we observed a confident interface between KCR1^WT^ and KCS9 within a KCR1‐KCS9‐ECR‐PAS2 tetramer AF3‐folded in the presence of FA and two NADPH ligands (Figure S2F,G). Notably, this included the intermediary face of the KCR1^WT^ protein (Figure 2D; Figure S2G), specifically the positively charged amino acids immediately adjacent to the site of the *kcr1‐2* mutation (Figure S2H). We interpret these results with caution, given the inability of AF3 to predict the effect of missense mutations on protein structure (Pak et al., 2023). Nonetheless, the data lend themselves to the speculation that attenuation of the KCR1–KCS9 interaction might be a promising hypothesis for further biochemical testing and might explain the viability of *kcr1‐2*.

### PIN polarity and trafficking are compromised in *kcr1‐2* roots

The availability of a viable mutant of the *KCR1* gene allowed us to investigate its role during post‐embryonic development. Defects in various components of VLCFA biosynthesis were previously shown to affect PIN protein polarity and the establishment of asymmetric auxin distribution (Ito et al., 2021; Roudier et al., 2010). It is also known that functional polar targeting and endocytosis require VLCFA biosynthesis, as exemplified by the formation of the cell plate during cytokinesis (Bach et al., 2011), polar sorting of PIN2 (Ito et al., 2021) and selective aggregation of auxin carriers AUX1 and PIN1 (Markham et al., 2011). Hence, equipped with the viable *kcr1‐2* mutant, we tested whether KCR1 might regulate the endocytic trafficking of PIN auxin transporters.

**Figure 4:**
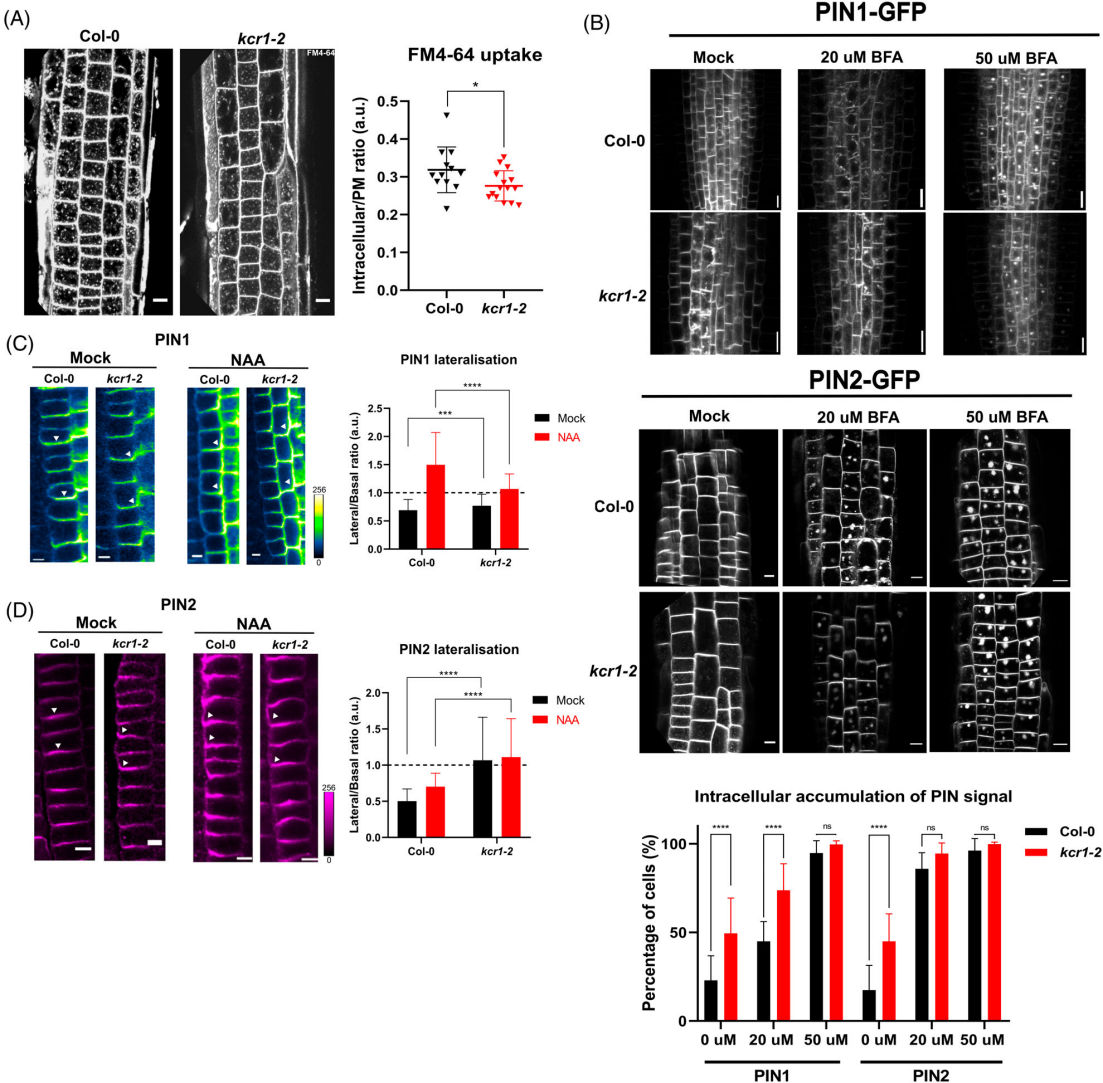
PIN endocytosis and membrane trafficking in *kcr1‐2* roots. (A) Representative images of *Arabidopsis* root epidermal cells treated with the FM4‐64 endocytic tracer dye. The *kcr1‐2* mutant shows reduced membrane uptake (Mann–Whitney test: *P* = 0.0365). For WT – *n* = 12 independent seedlings, 459 cells. For *kcr1‐2* – *n* = 15 independent seedlings, 405 cells. (B) Reduced BFA sensitivity of PIN1 and PIN2 trafficking in the *kcr1‐2* mutant. The chart quantifies cells showing intracellular PIN signal accumulation. Cells in the vasculature (PIN1) or epidermis (PIN2) were scored for existence of intracellular accumulation of GFP signal. Data are shown as a percentage of cells showing intracellular PIN1/2‐GFP signal as a part of all cells observed. Between 10 and 20 seedlings and between 200 and 300 cells per genotype were analyzed. Experiment was repeated three times with similar results. (C, D) Immunolocalization of PIN1 in the endodermis of WT and *kcr1‐2* roots (C) after 1‐h treatment with mock (DMSO) or 10 μM naphthalene acetic acid (NAA). Arrowheads indicate PIN polarity. The chart quantifies PIN lateralization as a signal ratio between lateral and basal cell membranes. (D) Immunolocalization of PIN2 in root cortex cells. (B–D) Two‐way ANOVA with Sidak's multiple comparison test (ns, *P* > 0.05; *P < 0.05; ***P = 0.0010; ****P < 0.0001). Scale bars: A, B – 10 μm; C, D – 5 μm. All bar plots indicate mean ± SD.

When treated with the lipophilic dye FM4‐64, which serves as a direct endocytic tracer (Jelínková et al., 2010; Johnson et al., 2020), *kcr1‐2* roots showed a decreased net internalization rate compared with WT (Figure 4A) indicating defects in endocytosis or endocytic trafficking. Next, we treated the WT and mutant roots with different concentrations of the trafficking inhibitor Brefeldin A (BFA) (Geldner et al., 2001) and observed intracellular accumulation (so‐called BFA bodies) of PIN1‐GFP and PIN2‐GFP in the stele and epidermal cells, respectively. The *kcr1‐2* mutant cells showed saturated BFA body formation already at lower BFA concentrations (Figure 4B) indicating increased BFA sensitivity. However, most strikingly, approximately 50% of the observed *kcr1‐2* cells show intracellular PIN aggregations without any treatment (Figure 4B). A similar accumulation of PIN1 without any BFA treatment was previously observed in *pas1* and *pas2* mutants defective in VLCFAs biosynthesis (Bach et al., 2011; Roudier et al., 2010). This observation confirms that VLCFA biosynthesis is vital for the functioning of the endomembrane system and trafficking of cargoes, such as PIN auxin transporters. However, it should be noted that the intracellular signal of PINs, in particular PIN2, in the BFA bodies likewise originates from the newly synthesized PINs (Jásik et al., 2016; Narasimhan et al., 2021).

A fundamental feature of PIN proteins is their polar subcellular distribution. Sphingolipids have been suggested to be involved in the polar sorting of PIN2 (Ito et al., 2021) and the disruption of very‐long‐acyl‐chain sphingolipids leads to deficiencies in asymmetric auxin accumulation (Ito et al., 2021; Markham et al., 2011). To probe PIN polarity and targeting, we performed immunolocalization with anti‐PIN1 and anti‐PIN2 antibodies in *kcr1‐2* roots, as described (Karampelias et al., 2018; Sauer et al., 2006). In WT roots, external application of the auxin analog naphthalene acetic acid (NAA) induces shifts in PIN polarity and leads to lateralization of these proteins (Hajný et al., 2022). Under NAA treatment, PIN1 re‐localizes to the inner and PIN2 to the outer cell sides of the endodermis and cortex root cells, respectively (Figure 4C,D). In contrast, in the *kcr1‐2* roots, both PIN1 and PIN2 showed less pronounced polar localization even without auxin treatment (Figure 4C,D). Following NAA treatment, *kcr1‐2* roots showed almost no changes in PIN1 and PIN2 positioning (Figure 4C,D) in contrast to WT. Furthermore, the PIN2 pattern at the PM in *kcr1‐2* plants shows a grainy distribution (Figure 4D). Interestingly, it has been shown that a mutation in the *STEROL МЕТHYLTRANSFERASE1* gene, which leads to abnormal membrane composition, results in PIN2 being more laterally than basally localized in vascular cells (Willemsen et al., 2003). These observations are in line with the stronger lateralization of PIN2 we present in this work.

To better understand the link between membrane dynamics and PIN2 localization, we performed fluorescence recovery after photobleaching (FRAP) experiments on PIN2‐GFP in the *kcr1‐2* seedling roots. We observed that the PIN2‐GFP signal is recovered more quickly in the *kcr1‐2* plants (Figure S4A). It is possible that the normal confinement of PIN protein clusters is lost in the mutant due to the altered membrane composition and that the lateral diffusion of PIN2 is less limited.

A fundamental but largely elusive concept in cell biology is that of trafficking specificity. Many mutants of trafficking‐related proteins display highly pleiotropic phenotypes, including the trafficking and polarity of PINs, but it is not clear whether these are primary or secondary defects. *Bona fide* PIN polarity regulators should exhibit trafficking specificity. We thus tested whether *kcr1‐2*'s effect on PIN polarity is specific or whether it represents a more general case of globally deranged cell polarity. To this end, we assessed another polar cargo: SOSEKI proteins. Interestingly, these showed normal expression and polarity in *kcr1‐2* roots (Figure S5A), without the grainy pattern of PINs in the mutant roots. SOSEKI proteins contain DIX domains and recruit ANGUSTIFOLIA to a complex, which results in a polymerization‐dependent maintenance of cell polarity (van Dop et al., 2020; Yoshida et al., 2019). The normal polarity of SOSEKI proteins in *kcr1‐2* roots suggests that intrinsic cell polarity remains intact.

Overall, these observations confirm a crucial and a specific role of KCR1 in the trafficking and polarity of PIN auxin transporters.

### Role of broadly expressed, ER‐localized KCR1 in abiotic stress responses

**Figure 5:**
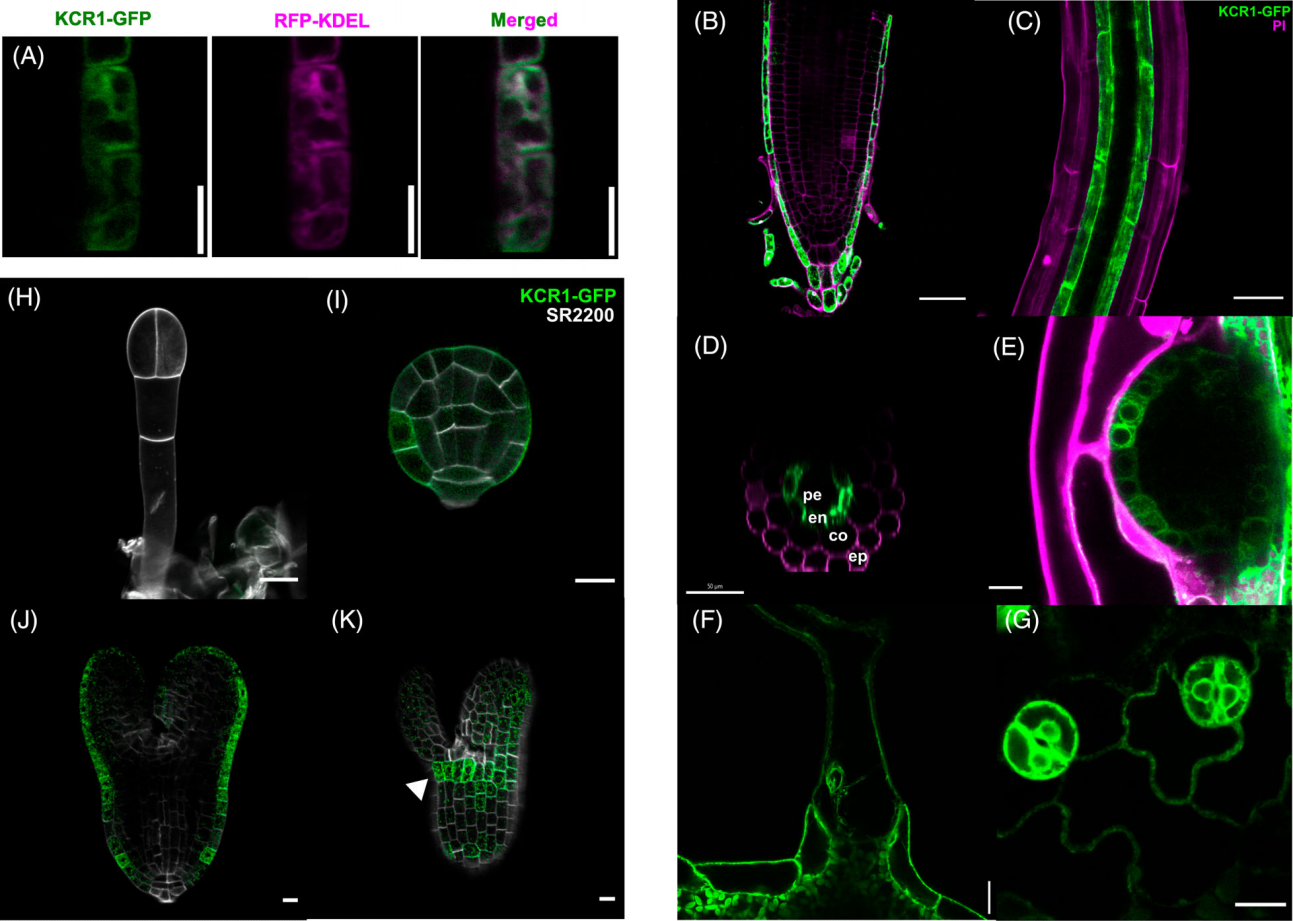
Expression pattern of KCR1‐GFP. (A) Co‐localization of KCR1‐GFP (green) with an ER marker RFP‐KDEL (magenta) in root epidermal cells. (B–G) Post‐embryonic KCR1 expression pattern. (B) Epidermis and lateral root cap. (C) Strong signal in the endodermis, (D) Transverse root cross‐section showing endodermal signal confinement, (E) Periphery of emerged lateral roots. (F) Trichome basal cells. (G) Stomatal guard cells. Green – GFP, magenta – propidium iodide (PI). Pe – pericycle; en – endodermis; co – cortex; ep – epidermis. (H–K) Expression pattern of KCR1 in embryos: (H) Lack of expression at the 2‐cell stage. (I) First expression at 32‐cell stage in the protoderm (green). (J) Expression is confined to protoderm in the late heart to (K) torpedo stage; Note a belt‐like pattern around the embryonic meristem (white arrowhead). Green – KCR1‐GFP. White – Renaissance stain SR2200. Scale bars:B–D – 50 μm, E – 25 μm, F – 20 μm, G – 10 μm. H–K – 10 μm.

We analyzed the *KCR1* expression pattern to gain additional insight into how its tissue‐specific activity might play a role in different developmental programs. Our qPCR analysis has revealed that there is no change in *KCR1* transcript levels in the *kcr1‐2* mutant as compared with WT (Figure S5C), further implying that the defects in *kcr1‐2* plants are manifested at the protein level. This led us to analyze the expression of the KCR1 protein itself. To this end, we generated a translational *pKCR1::KCR1‐GFP* fusion, which was functional as demonstrated by the full complementation of the *kcr1‐2* mutant (Figure 1E). The signal is ER‐associated (Figure 5A), as previously established (Batsale et al., 2021). The KCR1 in the primary root was enriched in the lateral root cap (Figure 5B). Other post‐embryonic tissues with strong KCR1 expression are the trichome basal cells (Figure 5F), which could explain the bent and malformed trichome defect observed in the *kcr1‐2* plants (Figure S5D) and KCR1 RNAi lines (Roudier et al., 2010). Stomatal guard cells likewise show a strong KCR1‐GFP signal (Figure 5G), which could be the reason for the mentioned hypersensitivity of *kcr1‐2* plants to increased irrigation.

Given the role of stomatal development and closure in stress responses, we tested whether *kcr1‐2* plants possess an altered response to abiotic stress. The *kcr1‐2* seedlings grown on medium containing sodium chloride and sorbitol show increased sensitivity to osmotic stress (Figure S6C). This observation is in line with previous findings (Joubès et al., 2008) showing that transcripts of VLCFA‐related genes, including *KCR1*, accumulate under exposure to NaCl, dehydration, and mannitol.

These findings highlight the importance of the broadly expressed KCR1 and downstream VLCFAs in a wide array of developmental processes and physiological functions.

### KCR1 expression and defective PIN1 polarity and patterning in *kcr1* embryos

In the embryos, the first instance when the KCR1‐GFP signal appeared was around the 32‐cell stage in the protodermal cells (Figure 5H,I). This timing of expression could be directly linked to the arrest of *kcr1‐1* embryos at the globular stage (Beaudoin et al., 2009). Later in embryogenesis, KCR1‐GFP expression remains restricted to the protoderm (Figure 5J). Interestingly, we observed that KCR1‐GFP is expressed in a striated manner in the cell layer below the embryonic SAM (Figure 5K). This could imply that KCR1 plays a role in restricting SAM overproliferation. This would be supported by extranumerary SAMs in the *kcr1‐2* (Figure 1A,B; Figure S1) and SAM overproliferation in the related *pas2* mutants (Nobusawa et al., 2013).

**Figure 6:**
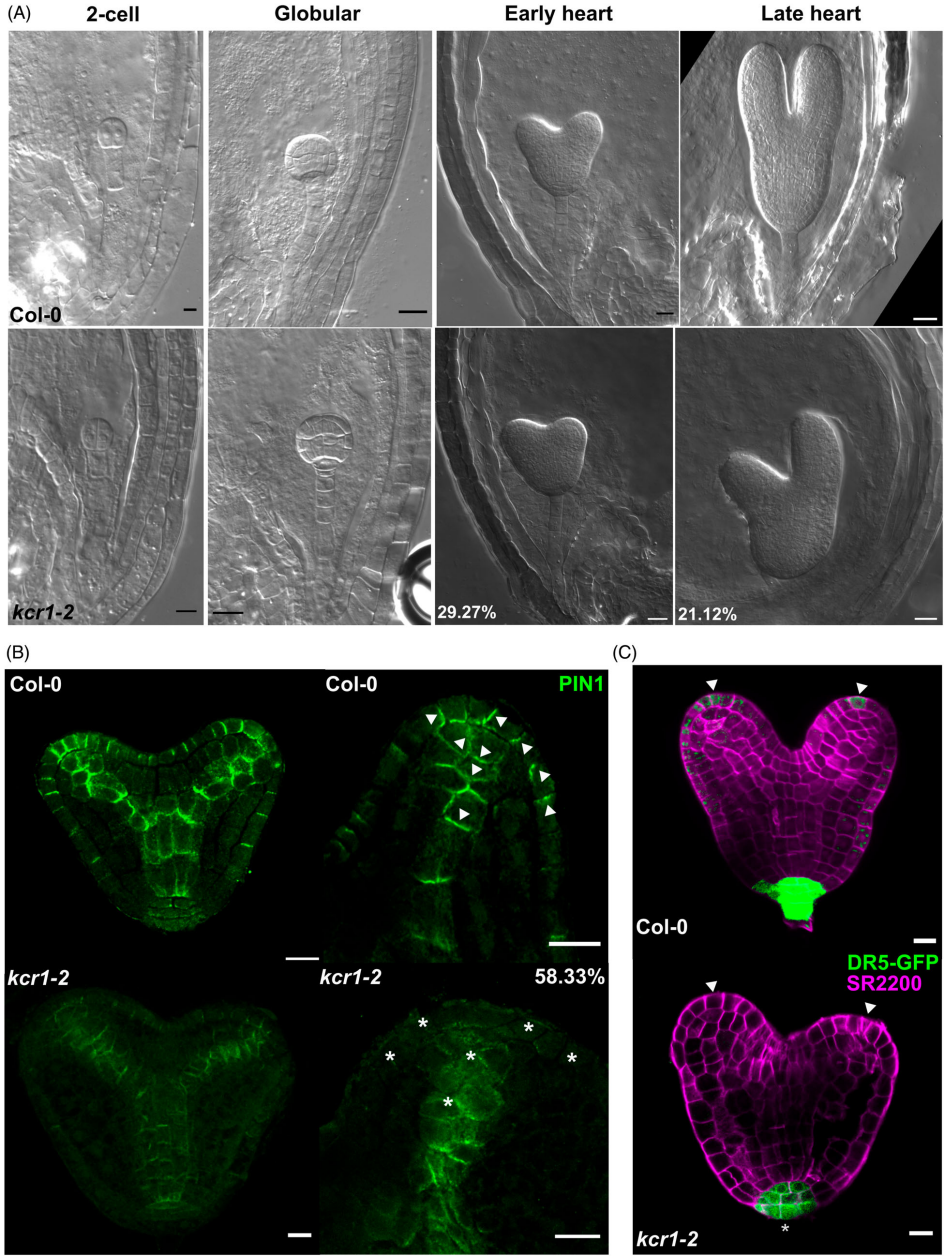
Auxin‐related embryonic defects in *kcr1‐2*. (A) DIC images of WT and *kcr1‐2* embryos. Phenotypic penetrance is indicated by percentages. The first *kcr1‐2* phenotype appears at the early heart stage. (B) Immunolocalization of PIN1 (green) in early‐heart‐stage embryos of the indicated genotypes. Arrowheads indicate the WT polarity of PIN1 in the protoderm and vasculature. Asterisks indicate the absence of signal and defective polarity in the cotyledon primordia of *kcr1‐2* embryos. The percentage indicates phenotypic penetrance. (C) *DR5rev::GFP* expression pattern in embryos of the indicated genotypes. Note the weaker DR5 signal at the root pole of *kcr1‐2* embryos (asterisk) and the absence of DR5 signal in cotyledon primordia (arrowheads). *DR5rev::GFP* (green), Renaissance SR2200 (magenta). Scale bars: A – 2‐cell stage 5 μm, rest – 10 μm; B, C – 10 μm.

Previous research on the role of VLCFAs in embryogenesis has revealed their crucial role in embryo patterning (Roudier et al., 2010). Nonetheless, a more precise role of KCR1 in embryogenesis was unknown due to early embryo lethality (Beaudoin et al., 2009). We found no defects in the *kcr1‐2* mutant from the first zygotic division up to the transition stage (Figure 6A), which are also stages largely characterized by the lack of *KCR1* expression. We observed first patterning defects at the early heart stage (Table S2), concurrent with the initiation of the cotyledon primordia (Mansfield & Briarty, 1991). The heart stage embryos were crooked or bent, and the patterning of the cotyledon primordia led to a blunted‐appearing apex of the embryos (Figure 6A). These embryonic phenotypes are similar to those that have been previously reported in mutants of other components of VLCFA biosynthesis (Faure et al., 1998; Roudier et al., 2010).

The apical patterning of the embryo and the emergence of cotyledons are developmental processes controlled by local auxin maxima resulting from the polarized localization of PIN1 auxin transporters (Benková et al., 2003; Friml et al., 2003). Therefore, we were interested in whether the cotyledon primordia defects are accompanied by a defective PIN polar targeting. To this end, we performed PIN1 immunolocalization in the early‐heart‐stage *kcr1‐2* embryos. In the majority of *kcr1‐2* embryos' and in all defective cotyledons, PIN1 expression level and polarity were defective (Figure 6B). In the WT protoderm cells, the PIN1 orients toward the future apices of the cotyledons (Figure 6B), but notably, the signal of PIN1 in the *kcr1‐2* protoderm cells was either very low or non‐existent. In the WT pre‐vasculature cells, PIN1 is oriented basally, toward the root pole (Figure 6B). However, the *kcr1‐2* PIN1 in the same cells shows much weaker basal polarity. The diffuse and partially intracellular signal of PIN1 (Figure 6B) is strikingly similar to the localization of PIN1 and PIN2 we observed in the root (Figure 4C,D).

Since the altered PIN targeting typically leads to defective asymmetric auxin distribution, we analyzed the pattern of the transcriptional auxin response marker *DR5rev::GFP* (Friml et al., 2003) in the *kcr1‐2* embryos. As expected, the defects in the DR5 pattern in the *kcr1‐2* embryos were correlated with the defects in the PIN polarity from the heart stage onwards. In the WT heart stage embryo, there is a strong root pole signal, with a weaker signal at the cotyledon primordia apices. On the contrary, in the *kcr1‐2* embryos, there is no signal in the cotyledons and the DR5 root pole signal is visibly weaker (Figure 6C). The absence of the DR5 signal at the cotyledon primordia apices may be a consequence of PIN1 defects in the protoderm. The lower DR5 signal in the root pole is likely due to the weak basal orientation of PIN1 in the pre‐vascular cells of *kcr1‐2* embryos.

Overall, we identified embryonic patterning defects of *kcr1‐2* that correlate with, and are likely the consequence of defective PIN1 positioning and the resulting defective asymmetric auxin distribution.

### KCR1 expression and defective PIN2 polarity and auxin homeostasis in *kcr1* primary roots

**Figure 7:**
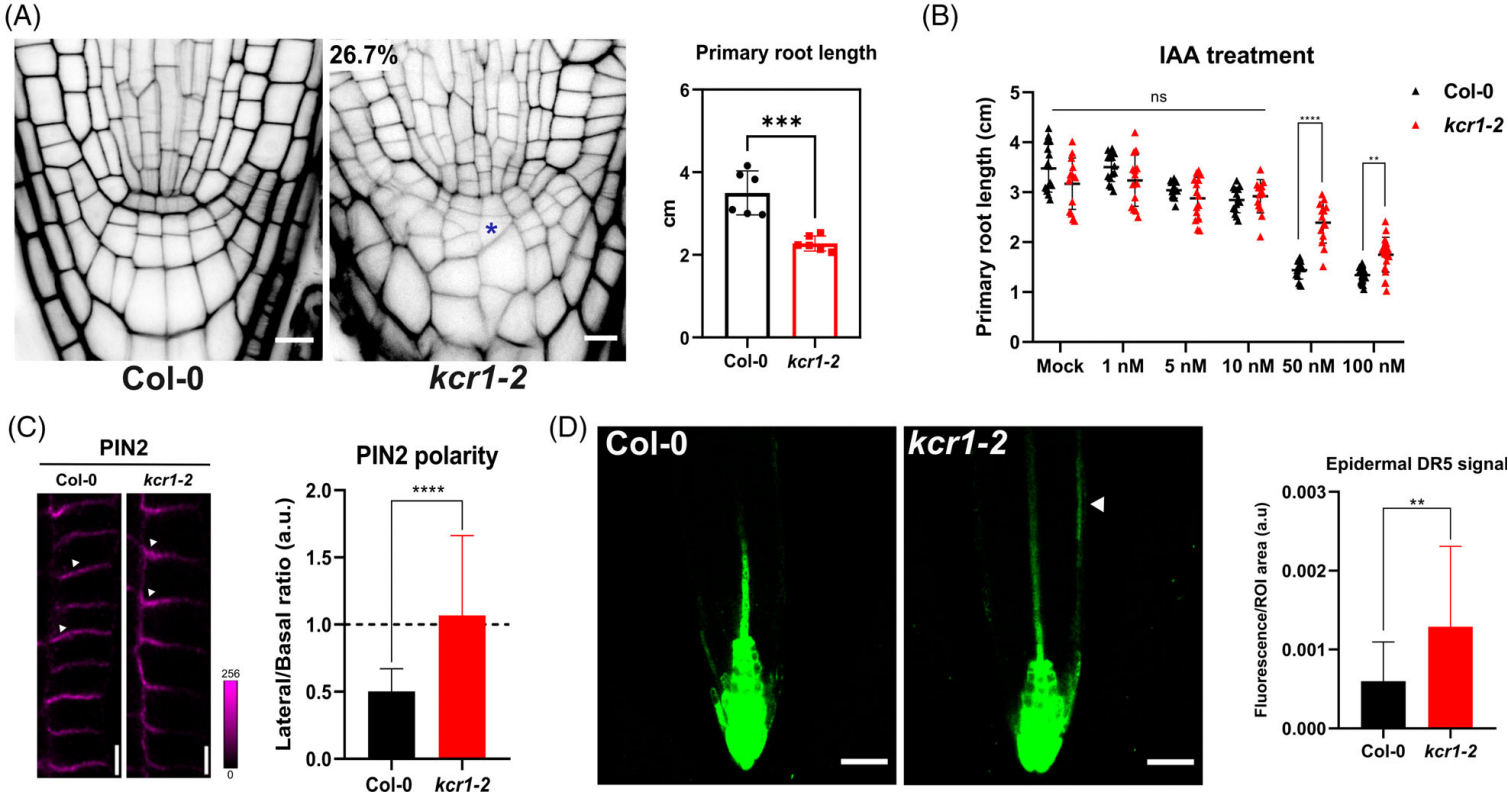
Auxin‐related defects in the *kcr1‐2* primary root. (A) Representative images of 5‐day‐old WT and *kcr1‐2* root tips (left). Asterisk indicates defective cell division planes in the columella. Percentage denotes phenotypic penetrance. Propidium iodide (PI) was used to stain cell walls. The chart (right) quantifies primary root length for WT and *kcr1‐2*. (B) Quantification of primary root length in response to increasing auxin (indole‐3 acetic acid, IAA) concentration. Two‐way ANOVA with Sidak's multiple comparison test (ns, *P* > 0.05; **P = 0.0023, ****P ≤ 0.0001). Median ± SD. (C) Immunolocalization of PIN2 in root cortex cells. Wild‐type PIN2 localizes to the base of cortex cells, whereas *kcr1‐2* roots show weak lateralization (white arrowheads). The chart quantifies PIN2 lateralization as a signal ratio between lateral and basal cell membranes. Mann–Whitney test (****P < 0.0001). (D) The *kcr1‐2* mutant shows increased DR5 signal in the root tip (Mann–Whitney test: **P = 0.0065). Arrowhead indicates DR5 signal extension to epidermal tissues. Scale bars: A – 10 μm; C – 5 μm; D – 50 μm.

In primary roots, KCR1‐GFP showed stronger expression in the lateral root cap (Figure 5B). Accordingly, *kcr1‐2* plants have shorter primary roots but normally developing cortex/endodermis and lateral root cap/epidermis initial cells (Figure 7A) with occasional defective divisions in the columella cells and columella stem cells (in about 27% of roots) as well as overall smaller and rounded root tips (Figure 7A). The *kcr1‐*2 roots during growth showed decreased sensitivity to auxin (Figure 7B; Figure S5B), indicating defective auxin transport which could be the consequence of the observed defects in root PINs. Our immunolocalization approach shows that PIN2 in *kcr1‐2* cortical cells shows defects (Figure 7C). Accordingly, the auxin response marker DR5 showed a stronger epidermal signal in the roots of *kcr1‐2* mutants (Figure 7D) indicating potentially defective auxin transport from these cells. This is in line with the observed defective PIN2 polarity and disrupted trafficking.

To further analyze the effect of the *kcr1‐2* mutation on the auxin in the primary root, we measured the DR5 signal in the QC and columella cells as previously described (Gelová et al., 2021) and observed a lower signal in the *kcr1‐2* roots (Figure S4C). Furthermore, measurements of the PIN2‐GFP signal in the *kcr1‐2* root cells show lower levels of fluorescence in the mutant roots (Figure S4B). Additionally, we performed a qPCR analysis of different auxin‐related genes (Figure S4D) and found no significant change either in the transcripts of auxin biosynthesis genes or in the AUX1 auxin importer. However, the expression levels of *PIN1* were significantly lower in the *kcr1‐2* mutant (Figure S4D), further strengthening the idea of a PIN‐specific effect of the *kcr1‐2* mutation.

### KCR1 expression and defective PIN polarity and development in *kcr1* lateral roots

The KCR1 is also expressed in the root endodermis (Figure 5C,D). This is consistent with the expression of other VLCFA biosynthetic enzymes in the same layer (Shang et al., 2016) and their potential role in lateral root initiation (Roudier et al., 2010; Shang et al., 2016) as well as emergence (Shang et al., 2016; Trinh et al., 2019). We observed that KCR1 is highly expressed in the boundary cells of lateral root primordia (LRP) and emerged lateral roots (Figure 5E).

**Figure 8:**
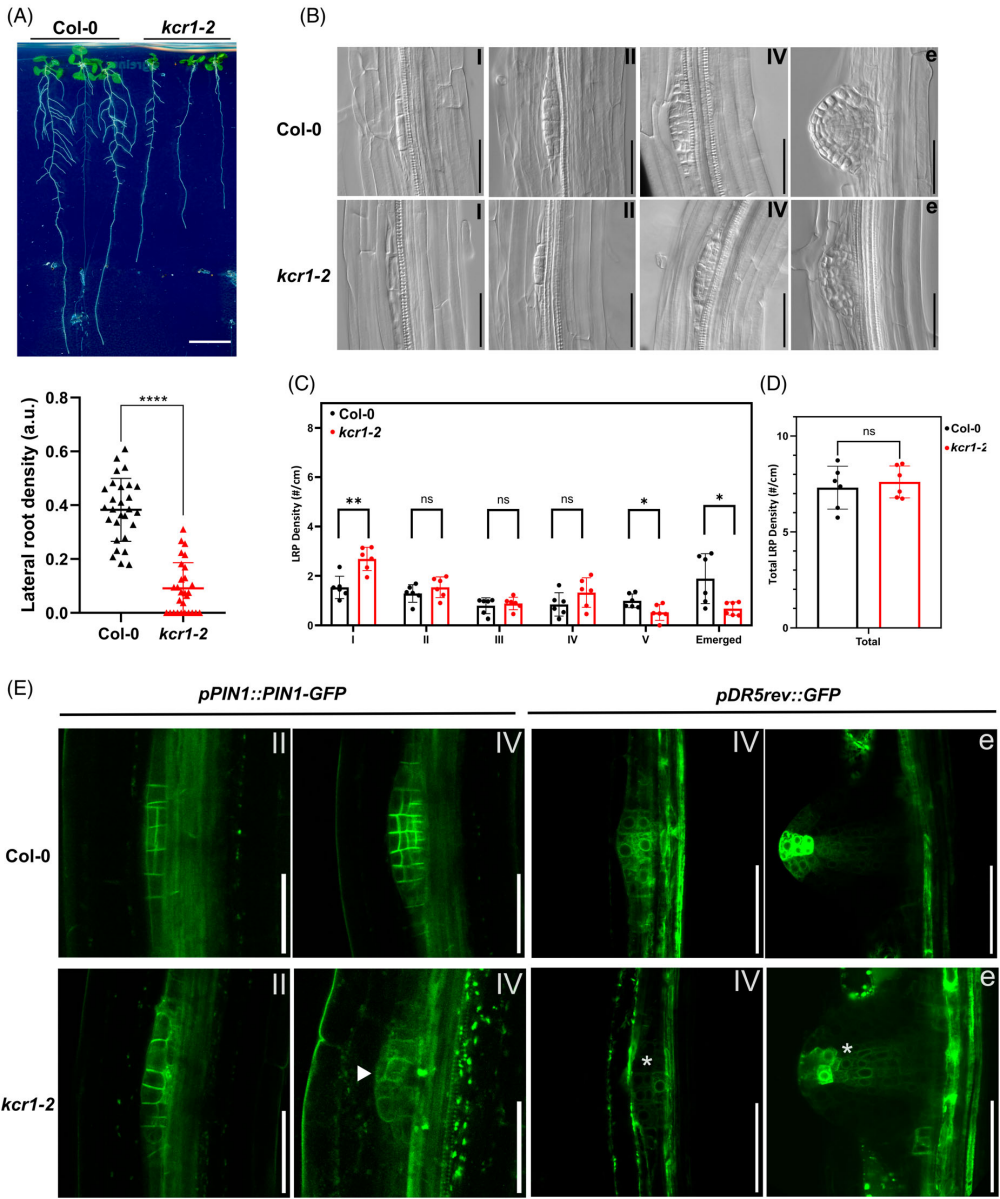
Auxin‐related defects in *kcr1‐2* lateral roots. (A) Upper panel – representative image of 12‐day‐old WT and *kcr1‐2* seedlings showing the emergence of lateral roots. Lower panel – quantification of lateral root density expressed as a ratio of emerged lateral roots to primary root length. Mann–Whitney test (****P‐value ≤ 0.0001). Mean ± SD. (B) Cleared lateral root primordia (LRP). Representative images of individual stages are marked with roman numerals.e‐– emerged lateral root. (C) Stage profile of lateral roots. Data depict the number of lateral roots per cm of primary root length. (D) Total LRP density (*t*‐test, ns, *P* > 0.05). (E) Representative expression pattern of PIN1‐GFP in stages II and IV LRPs (left panels). Note defective cell shape in *kcr1‐2* as well as low PIN1‐GFP signal and polarity in stage IV (arrowhead). Representative expression of *DR5rev::GFP* in LRP stage IV and emerged lateral roots between genotypes (right panels). Note lower *DR5rev::GFP* signal in *kcr1‐2* lateral root primordia and emerged lateral root (asterisks). Scale bars:A‐– 10 mm; B, E – 50 μm.

The *kcr1‐2* plants form visibly fewer emerged lateral roots (Figure 8A). When we analyzed the lateral root formation stages in detail, we observed that at all stages, the LRPs show disordered and defective division planes (Figure 8B). In *kcr1‐2*, the LRP edges at stages I and II showed seemingly detached cells (Figure 8B). At the later stages, the LRP and emerged roots showed overall shape defects. Whereas the total number of LRPs was approximately equal between *kcr1‐2* and WT, we observed that there were more stage I LRPs in *kcr1‐2* (Figure 8C). This suggests that there is an increase in the pericycle tissue proliferation, mirroring the similar overproliferation in the aerial organs (see Figure 1; Figure S1). Considering that KCR1 is expressed in the endodermal cell layer, VLCFAs produced in this tissue might inhibit the competence of the pericycle for lateral root initiation, as suggested previously (Shang et al., 2016). In the *kcr1‐2* mutant, this inhibition might be disturbed, leading to an increase in LRP initiation. Looking at the LRP stage profile, there is no difference in the frequency of LRPs between genotypes in stages II–IV (Figure 8C). However, there is a decrease in stage V and emerged lateral roots (Figure 8C), either suggesting that the mutation affects the transition between stages IV and V or that the emerging *kcr1‐2* lateral roots have difficulty penetrating the overlaying tissue layers. The distorted shape of the emerged lateral roots supports the latter. An abnormal profile of LRP stages was likewise observed in the *puchi1* mutant (Trinh et al., 2019).

Auxin gradients, resulting from the polarized action of PIN1 and other PINs, are known to mediate lateral root initiation, cell division, and expansion in LRP (Benková et al., 2003). We therefore analyzed the pattern of PIN1 localization by observing PIN1‐GFP in the lateral roots of *kcr1‐2*. Our observations clearly show defects in the expression, distribution, and polarity of PIN1 (Figure 8E). As PIN1 polarity defects commonly lead to defects in auxin distribution (Zhang et al., 2010), we analyzed DR5 auxin response marker expression in *kcr1‐2* lateral roots. We observed that the DR5 maxima at the LRP tip during emergence are weaker (Figure 8E). This phenomenon could be directly related to the disrupted PIN1 localization and explain the LRP emergence defects since the LRP tip auxin maximum has been linked to the loosening of tissue around the LRP facilitating its emergence (Swarup et al., 2008).

Thus, again as seen in embryos and primary roots, during LRP development, defective PIN polarity and auxin response distribution in *kcr1‐2* mutants correlate with corresponding developmental defects.

## DISCUSSION

### VLFCAs play an important role in plant development

VLCFAs are crucial components of plant physiology and development (Haslam & Kunst, 2013). There is an established link between VLCFAs and cell division as well as differentiation (Roudier et al., 2010). Disruption of VLCFA biosynthesis in plants leads to severe growth and patterning defects (Faure et al., 1998), presumably due to the importance of VLCFA for vesicle trafficking (Markham et al., 2011). VLCFAs are components of cuticular waxes, suberin, and sphingolipids (Haslam & Kunst, 2013). VLCFA‐containing sphingolipids have been proposed to define specific compartments for polar targeting of proteins (Markham et al., 2011), including PIN auxin transporters, which are crucial for most development (Friml, 2022; Luschnig & Friml, 2024). Genetic and biochemical studies have led to a model in which a heterotetrameric protein complex mediates VLCFA elongation in the ER (Haslam & Kunst, 2013). One component of this complex is the β‐Ketoacyl‐CoA Reductase 1 (KCR1). The KCR1 activity is essential for plant development, as the T‐DNA insertional mutant *kcr1‐1* is embryo lethal (Beaudoin et al., 2009). The identification of the viable *kcr1* allele (*kcr1‐2*) allowed us to study the scope and mechanism of KCR1 function in plant development.

### Developmental defects of *kcr1‐2* as a consequence of altered PIN polarity and auxin distribution

The kcr1‐2 mutation does not seem to affect the KCR1 catalytic site or binding to its ligand. However, since the surface potential of the kcr1‐2 protein is altered, it is possible that the normal protein–protein interactions of the VLCFA elongation complex are disrupted (Haslam & Kunst, 2013) leading to attenuated VLCF synthesis. The *kcr1‐2* plants exhibit defects at all developmental stages, confirming the crucial role of VLCFA biosynthesis in overall patterning and plant morphology.

It has been previously reported that mutants in VLCFA biosynthesis possess affected polarity of PIN auxin transporters (Bach et al., 2011; Roudier et al., 2010). The PIN polar deposition relies heavily on dynamic subcellular vesicle trafficking (Adamowski & Friml, 2015). The limitation of lateral diffusion in the membranes leads to PIN proteins being confined to a polar disposition (Glanc et al., 2021). Furthermore, in the short time frame used in our photobleaching approach, the dynamics of PINs would almost entirely depend on lateral diffusion (Langowski et al., 2016; Men et al., 2008). In the FRAP experiments on the *kcr1‐2* mutant, we observed that the lateral diffusion of PIN2 is increased. This phenomenon is possibly a consequence of an altered membrane composition, further underlying the importance of inherent membrane structure for PIN protein stability. Having a viable *kcr1* allele, we focused on analyzing the role of KCR1 and VLCFAs in PIN trafficking and polarity. Indeed, the polarity and constitutive recycling of PIN1 are disrupted in the *kcr1‐2* mutant as manifested by diminished polar distribution, increased intracellular accumulation, and a defect in auxin‐induced relocation of these proteins. The *kcr1‐2* mutation seems to affect PINs somewhat specifically as another polar cargo, the SOSEKI proteins (Yoshida et al., 2019) are unaffected.

Since PINs and their polarity mediate intercellular auxin flux (Luschnig & Friml, 2024), these defects are associated with perturbations in asymmetric auxin distribution, as observed in embryos, primary roots, and LRP. Our measurement of auxin‐related genes' transcripts strongly indicates that auxin transport, rather than auxin biosynthesis, is affected in the *kcr1‐2* mutant. Notably, transcript levels of AUX1 auxin importer were not affected in the *kcr1‐2* mutant, consistent with normal AUX1 disposition in the membrane of the *pas1‐3* mutant (Roudier et al., 2010). These results further strengthen the idea that PINs are specifically affected by the *kcr1‐2* mutation. As the expression of *PIN* genes is auxin‐inducible (Vieten et al., 2005), these results could indicate a potential existence of a feedback loop between low auxin levels and low PIN expression rates. Future research will address the connection between the VLCFA biosynthesis and overall auxin dynamics in plants.

Consequently, we observe that the auxin‐associated developmental processes are disturbed in *kcr1‐2* mutants, including embryogenesis, root meristem activity, auxin‐sensitive root growth, lateral root organogenesis, and control of SAM proliferation.

We argue that strong developmental defects in *kcr1‐2* mutants could be attributed to the defects in PIN polarity and auxin distribution, supporting a key and rather specific role of VLCFAs in the regulation of PIN trafficking and polarity. PIN1 seems less redundant in generating auxin gradients for aerial organogenesis (Luschnig & Friml, 2024), implying a link between the defective polarity and trafficking of PIN1 and the strong defects in apical patterning of *kcr1‐2*.

### Extranumerary SAMs of *kcr1‐2* may be a result of meristem cell regulatory network disruption

A conspicuous phenotype of *kcr1‐2* is the appearance of extranumerary SAMs. This phenotype highlights the importance of VLCFA in the establishment and limiting of meristematic identity. Some VLCFA‐related regulators, such as PUCHI, a transcription factor of the AP2/ERF family, play a role in the correct spatio‐temporal regulation of the expression of key molecular players during meristem formation (Bellande et al., 2022).

Functional VLCFA biosynthesis has also been shown to be an important factor in limiting cell proliferation in SAMs (Nobusawa et al., 2013). This notion is strengthened by the observation that a *pas2* mutation rescues the *shoot meristemless* (*stm*) phenotype (Harrar et al., 2003). Therefore, when VLCFA metabolism is disrupted, meristem‐related genes are ectopically expressed, and the meristematic tissue proliferates disproportionately. The supernumerary, ectopic meristems observed in *kcr1‐2* may be the extreme manifestation of a similar disruption of a VLCFA‐dependent regulatory network inhibiting meristematic identity and cell proliferation. Interestingly, research on *Medicago* implies that biosynthesis of VLCFAs in outer layers of SAMs directly impacts plasma membrane integrity and therefore affects the localization of PIN1, playing a role in leaf primordia development, further linking VLCFA function with auxin transport (Wang et al., 2023).

### Local KCR1 expression defines boundaries with a potential non‐cell autonomous action of VLCFAs

Analysis of KCR1 expression revealed a typical pattern of expression at the boundaries of tissues and organs, including: (i) protoderm cells around the embryo; (ii) a belt‐like expression around the SAM; (iii) lateral root cap cells surrounding the distal root meristem; (iv) cells at the base of the trichomes; (v) endodermis encircling pericycle, where lateral roots initiate; and (vi) boundaries of developing LRP. Intriguingly, the developmental defects in the *kcr1‐2* mutant were not observed in these tissues where KCR1 was strongly expressed, but rather in the adjacent, more central tissues such as the overproliferating SAM; defective root apical meristem; proliferation‐like defects in the trichomes manifested by an increased number of papilli (Figure S5D); increased proliferation of the pericycle; and defective LRP. These consistent observations in our study have led us to hypothesize that KCR1 activity seems to be confined to a boundary, which is defined around tissues, such as lateral roots, trichomes, cotyledon primordia, or SAMs. The products of KCR1 activity—VLCFAs –may then act in a non‐cell autonomous manner and thus be important developmental cues regulating cell proliferation and differentiation of the aforementioned specialized cell populations.

Previously, it has been proposed that VLCFA‐containing ceramides mediate the determination of protoderm by acting as a putative signaling molecule that would interact with ATML1, a tissue differentiation factor (Nagata et al., 2021). Indeed, it has been shown that VLCFAs and their tissue‐specific biosynthesis act as positional signals for tissue differentiation in callus formation capacity in roots (Shang et al., 2016), as well as lateral root formation (Trinh et al., 2019), the latter process also being dependent on the activity of the VLCFA‐dependent transcription factor MYB93 (Uemura et al., 2023). Findings such as these imply that in the plant organism, there exists a tight regulatory network depending on the tissue‐specific biosynthesis of VLCFAs that act as positional signals regulating organogenesis via different genetic and physiological pathways.

## MATERIAL AND METHODS

### Plant material and growth conditions

In all experiments, *Arabidopsis thaliana* (L.) Col‐0 (NASC, The Nottingham Arabidopsis Stock Center; https://www.arabidopsis.info, N1092) was used as a WT. Previously published marker lines were used: *pPIN1::PIN1‐GFP* (Benková et al., 2003); *pPIN2::PIN2‐GFP* (Abas et al., 2006); *DR5rev::GFP* (Friml et al., 2003)*; p35S::RFP‐HDEL* (Nelson et al., 2007)*; pSOK1::SOK1‐YFP, pSOK2::SOK2‐YFP, pSOK3::SOK3‐YFP, pSOK4::SOK4‐YFP, pSOK5::SOK5‐YFP* (Yoshida et al., 2019)*; kcr1‐1* mutant was obtained from the Plant Functional Genomics Research Group of RIKEN Genomic Sciences Center (https://epd.brc.riken.jp/en/seed/activ, Ds transposon insertional line RATH12‐5282‐1‐G). The *ectopic shoot meristems (esm)* mutant plants were backcrossed with Col‐0 to remove the *pPIN3::PIN3‐GFP* background. The *esm* mutant plants were genotyped by observing the phenotype and Sanger sequencing. The relevant markers were introduced in the *esm* background by genetic crossing. *pKCR1::KCR1‐GFP* line was introduced in Col‐0 and *esm* backgrounds by floral dipping. Seeds were surface sterilized by chlorine gas, sown on 0.5× Murashige and Skoog (MS) medium supplemented with 1% (w/v) sucrose and 0.8% (w/v) phytoagar (pH 5.9), stratified at 4°C for 2 days, and then grown at 21°C with a long‐day photoperiod (16 h light/8 h dark). For dark treatment experiments, sown and stratified seeds were exposed to light for 6 h, covered with aluminum foil, and cultivated at 21°C for 4 days (hypocotyl growth and gravitropic response experiments) or 5 days (SAM analyses). For adult phenotypical analysis, plants in soil were cultivated at 22°C and 50% relative soil humidity.

### EMS mutagenesis and gene cloning

EMS mutagenesis has been done previously (Rakusová et al., 2019). Briefly, p*PIN3:PIN3‐GFP* in Col‐0 seeds was mutagenized by 0.3% MES. The *esm* mutant was identified in M2 and further confirmed in M3 according to its unique phenotypes. The responsible gene was successfully cloned by a combination of bulk segregant analysis and NGS sequencing. Map‐based primary mapping was performed following the standard method using an F2 population from crossing the *h* mutant with Ler. Whole‐genome sequencing was done as previously described (Rakusová et al., 2019). Briefly, the *esm* mutant was backcrossed with *PIN3:PIN3‐GFP*. About 60–80 mutant seedlings were selected from the F2‐segregating population for DNA isolation using the DNeasy Plant Kit from QIAGEN, which was then sent for whole‐genome sequencing (BGI, http://www.genomics.cn/en/index).

### Molecular cloning

To generate the *pKCR1::KCR1‐GFP* construct, Gibson Assembly (NEB) and Gateway Cloning (Invitrogen) were used: The full‐length promoter of *KCR1* was amplified from *Arabidopsis thaliana* Col‐0 genomic DNA using Phusion High‐Fidelity DNA Polymerase (NEB) and cloned into the pDONRP4P1r entry vector (Invitrogen) by Gibson Assembly using 2x HiFi Mix (NEB). The *KCR1* coding sequence (CDS) without the stop codon was amplified from Col‐0 cDNA. *KCR1* CDS and *GFP* were cloned by BP Clonase (Invitrogen) into pDONR221 and pDONRP2rP3 entry vectors, respectively. The three fragments were recombined into the destination vector PB7m34GW using LR clonase II (Gateway, Invitrogen). Cloning primers are provided in the Table S3. All vectors at every step were confirmed by Sanger sequencing. The floral dip method was used to transform *Arabidopsis thaliana* plants with *Agrobacterium tumefaciens* strain GV3101.

### Histological analyses

Visualization of embryo phenotype was performed as previously described (Vermon & Meinke, 1994). Siliques of different developmental stages were removed from plants, and ovules were dissected on a two‐sided adhesive tape under a stereomicroscope (Leica EZ4). The ovules were cleared in Hoyer's solution in the dark at room temperature, with varying times according to their developmental stages. The embryos were then observed under a microscope equipped with Nomarski optics (Carl Zeiss Axio Imager A2) and images were taken with the AxioCamI1.

### Lateral root density analysis

For lateral root density analysis, plates with 12‐dayday‐old seedlings were scanned using an Epson Perfection V370 Photo flatbed scanner. The number of lateral roots was counted for each seedling and divided by the length of the primary root. The lengths of the primary roots were measured in ImageJ using the SNT (Simple Neurite Tracer) add‐on.

### Hypocotyl analyses

For etiolated hypocotyl analyses, seedlings were grown in the dark for 4 days and scanned using an Epson Perfection V370 Photo flatbed scanner. Hypocotyl length was measured between the hypocotyl/root junction and the apex of the seedling in ImageJ using the SNT (Simple Neurite Tracer) plugin. For the hypocotyl gravitropic response (hypocotyl bending) experiments, seedlings were grown in the dark for 3 days and rotated 90°. After 24 h, the plates were scanned, and the bending angle was measured using the angle tool in ImageJ.

### Cotyledon analyses

For light‐grown cotyledon analysis, 5‐dayday‐old seedlings were scored for the cotyledon classes. To analyze the dark‐grown cotyledons, the seedlings were grown in the dark for 5 days. The etiolated seedlings were cleared as described previously (Malamy & Benfey, 1997). To prevent clotting, the roots of the seedlings were cut prior to clearing. The cleared seedlings were mounted in 50% glycerol and imaged using DIC optics on an Olympus BX53 microscope.

### qPCR analysis

Total RNA was extracted from excised 7‐day‐old seedling roots and shoots (for KCR1 transcript analysis) or 8‐day‐old seedling roots (for auxin‐related genes' transcripts analysis) using RNeasy® Plant Mini kit from QIAGEN according to the manufacturer's protocol. About 1 and 0.5 μg of RNA was used to synthesize cDNA (shoot and root respectively) using iScriptTM cDNA synthesis kit (BIO Rad). The analysis was carried out on a LightCycler 480 II (SW1.5.1 Version; Roche Diagnostics) with the SYBR Green I Master kit (Roche Diagnostics) according to the manufacturer's instructions. All PCR reactions were carried out with three biological and technical triplicates. Expression levels of target genes were quantified by specific primers that were designed using Quant Prime (Arvidsson et al., 2008) and validated by performing primer efficiency for each primer pair. The levels of expression of each gene were first measured relative to AT4G05320 (UBQ10) or to AT1G69960 (PP2A) and then to respective genotypes. Significant differences between the expression level of *KCR1* between WT and *kcr1‐2* were calculated using two‐tailed t‐test. For auxin‐related genes, one‐way ANOVA with Dunnett's multiple comparisons test was used. qPCR primers are provided in the supplementary information section (Table S3).

### Propidium iodide (PI) staining

For PI (Thermo Fisher Scientific, P3566) staining, 4‐dayday‐old seedlings were incubated in PI diluted to a final concentration of 10 mg L^−1^ in H_2_O for 5 min and washed once with H_2_O before imaging using inverted CLSM (Zeiss LSM800).

### FM4‐64 staining and analysis

For the analysis of FM4‐64 uptake, the previously described method was used (Johnson et al., 2020). 5‐dayday‐old seedlings were incubated in 6‐well plates containing 2 μM FM4‐64 dye (Invitrogen, T13320) diluted in liquid MS medium supplemented with 1% sucrose for 5 min at room temperature. The seedlings were then gently washed twice for 30 sec to 1 min in liquid medium without the dye. After the washing, seedlings were immediately transferred to the microscope slide (76 × 26mm; Assistent 42 406 020), mounted in liquid MS medium, and covered with a coverslip (24 × 50 mm, thickness 1.5; VWR #631–0147) and immediately imaged using an inverted CLSM (Zeiss LSM800). The FM4‐64 uptake was analyzed using a published MatLab script (Johnson et al., 2020). For both genotypes, between 12 and 14 seedlings were imaged, and between 400 and 450 cells were analyzed using the MatLab script.

### BFA treatment and analysis

Four‐day‐old seedlings were incubated in 24‐well plates containing liquid MS medium with Brefeldin A (BFA) at a final concentration of 50 μM for 1 h at room temperature on a platform shaker. As a control treatment, DMSO (Plant cell culture tested; Sigma‐Aldrich D4540) was used. After the treatment, the seedlings were mounted in the incubation solution and imaged immediately using an inverted CLSM (Zeiss LSM800). A small z‐stack was taken in the elongation zone of the root to observe the membrane and the inner parts of the cell. For each seedling, 15 cells in one cell file of a single z‐stack plane were analyzed to score intracellular signal. The intracellular signal of interest was the presence of intracellular fluorescent signal in vesicles (irrespective of size of the vesicles). Data were presented as the percentage of cells in which accumulation of intracellular fluorescent signal was observed relative to all cells analyzed.

### Immunostaining

Immunostaining on roots was performed with 3‐dayday‐old seedlings as described previously (Sauer et al., 2006). The primary antibodies used were rabbit anti‐PIN1 and anti‐PIN2, both diluted 1:1000 (v/v). The secondary antibody used was sheep anti‐rabbit conjugated with Cy3 (Sigma‐Aldrich, C2306), diluted 1:600 (v/v). Immunostaining on embryos was performed as described (Sauer et al., 2006) on Polysine slides (VWR, 631–0107). The primary antibody used was rabbit anti‐PIN1 diluted 1:1000 (v/v). The secondary antibody used was goat anti‐rabbit conjugated with Alexa Fluor 488 (ThermoFisher Scientific, A‐11008), diluted 1:600 (v/v).

### PIN lateralization

Three‐day‐old seedlings were incubated either with 10 μM naphtaleneacetic acid (NAA) or DMSO as a control for 4 h in liquid ½ MS medium. Subsequently, immuno‐staining using PIN1 and PIN2 antibodies was performed. Samples were imaged using a confocal microscope (Zeiss LSM800). The fluorescence intensity of Cy3 (excitation wavelength: 548 nm) was measured using Image J.

### PIN2 FRAP experiments

For FRAP experiments, 4 or 5 days old seedlings were mounted on slides with liquid ½ MS medium and imaged on the Zeiss LSM800 before bleaching. For the bleaching, we used the built‐in bleach function in the Zeiss ZEN Blue software. In short, we used combined 100% 488 nm and 30% 405 nm lasers for bleaching and afterwards performed time lapse imaging, acquiring an image every 5 sec. For the analysis, images from 30 sec steps were used, and fluorescence measurement was done in ImageJ.

### *DR5rev::GFP* analysis

Five‐day‐old seedlings were transferred from the solid ½ MS medium and mounted on slides with liquid ½ MS medium and imaged on a confocal microscope. All roots were imaged using the same laser power and digital gain settings, with caution not to induce a gravitropic response. Fluorescence intensity was measured in the epidermis, from the uppermost edge of the quiescent center cell to the upper edge of the microscopic image, and in the root apical meristem in all columellar cells from and including quiescent center cells until the root cap. The measured fluorescent intensity was divided by the area of the region of interest. Measurements of fluorescence were performed in ImageJ. *DR5rev::GFP* signal in embryos was analyzed by excising the embryos of the desired stage from the ovules in 0.1% counterstain solution (Renaissance Chemicals, SR2200) and imaging with a confocal microscope (Zeiss LSM800, 40x WI objective).

### Abiotic stress analysis

Five‐day‐old seedlings were transferred to solid ½ MS medium containing the treatment (100 mM Nacl, 200 mM sorbitol or mock treatment) and grown on vertically placed treatment plates for 7 days. Afterwards, the plates were scanned using an Epson Perfection V370 Photo flatbed scanner and analyzed in ImageJ.

### Electron microscopy

Four or Five‐day‐old dark‐grown seedlings were fixed in 4% PFA under vacuum at room temperature for 1 h. Afterwards, the seedlings were washed three times with 1× PBS for 15 min and then three times with dH_2_O for 15 min. Afterwards, the seedlings were dehydrated in a series of ethanol dilutions. They were stored for critical point drying in 100% ethanol. Once in 100% ethanol, the seedlings were dried with a critical point dryer (EM‐CPD3000, Leica Microsystems) and subsequently coated with gold using a sputter coater (EM‐ACE600, Leica Microsystems). The samples were finally imaged with a scanning electron microscope (FE‐SEM Merlin compact, VP, Carl Zeiss) at 5 kV using a secondary electron detector.

### Lipid extraction and analyses

Total fatty acids were measured after FAME preparation from 2 mg of lyophilized plant material followed by gas chromatography coupled with mass spectrometry (GC–MS) analysis as previously described (Li et al., 2006). Sphingolipids, Cers, hCers, GlcCers, and GIPCs were extracted from 2 mg of lyophilized plant material and analyzed with ultra‐high performance liquid chromatography (UPLC)‐electrospray ionization (ESI)‐tandem mass spectrometry (MS/MS) analyses (Tellier et al., 2014). The mass analyses were performed in the positive multiple reaction monitoring (MRM) mode. Chromatographic conditions, mass spectrometric parameters, and sphingolipid MRM methods were defined previously (Tellier et al., 2014).

### Structural modeling

Predictions of protein structures were performed using AlphaFold 3 (AF3) server (Abramson et al., 2024). Amino acid sequences or crystal structures for the analyses were downloaded from the Uniprot or the Protein Data Bank (PDB) databases, respectively, and individual Uniprot ID or PDB ID codes are detailed in the figure legends.

To create a superimposition of KCR1^WT^ and KCR1^kcr1–2^ monomer or dimer AF3 predictions, we used the'alig' command in PyMOL (The PyMOL Molecular Graphics System, Version 2.5.2, Schrödinger, LLC). To superimpose the crystal structure of the human 17β‐hydroxysteroid dehydrogenase type I with the KCR1^WT^ AF3 model, we employed the sequence‐independent'supe' command, which executes a structure‐based dynamic programming alignment with iterative refinement.

Coloring of AF3 structural predictions by pLDDT was achieved using a Pymol extension coloraf.py available from GitHub by Dr. Christina Balbin: https://raw.githubusercontent.com/cbalbin‐bio/pymol‐color‐alphafold/master/coloraf.py [retrieved 2025‐05‐22].

Maps of electrostatic surface potential were generated in Pymol using the APBS Electrostatics plugin (Baker et al., 2001).

Multiple sequence alignment was with the MUSCLE tool provided by EMBL EBI (Madeira et al., 2022) with default parameter settings. The alignment was analyzed with Jalview 2.11.2.2 (Waterhouse et al., 2009).

### Statistical analysis and data visualization

Statistical analyses and data visualization was performed using GraphPad Prism 8. Figures were assembled in Inkscape 1.2.

## Conflict of Interest

Authors declare no competing interests, financial or otherwise.

## Supporting information


**Figure S1.** Apical phenotypes and allelic test to identify the *esm* mutation.
**Figure S2.** VLCFA measurements in *kcr1‐2* mutants.
**Figure S3.** Predictions of the KCR1 structure.
**Figure S4.** Changes in PINs and auxin distribution in the *kcr1‐2*.
**Figure S5.** Analysis of *kcr1‐2* root and trichome phenotypes, *KCR1* gene expression.
**Figure S6.** Hypocotyl defects and abiotic stress sensitivity of *kcr1‐2*.

**Table S1.** Chromosome 1 mapping region annotation. We show gene identifiers, variant types and predicted impacts, associated transcripts and features, exon/intron context, HGVS nomenclature along with positional information.

**Table S2.** Summary of *kcr1‐2* embryonic phenotypes.

**Table S3.** Primers used in the study.

## Data Availability

The data that support the findings of this study are available on request from the corresponding author. The data are not publicly available due to privacy or ethical restrictions.
