# Supplementary material for: Biosynthesis of very Long‐chain fatty acids is required for Arabidopsis auxin‐mediated embryonic and post‐embryonic development
Source: Plant J. 2025 Aug 9;123(3):e70396. doi: 10.1111/tpj.70396 (PMC12335294; doi:10.1111/tpj.70396)
Supplement: Supplementary file 1 — Figure S1. Apical phenotypes and allelic test to identify the esm mutation. Figure S2. VLCFA measurements in kcr1‐2 mutants. Figure S3. Predictions of the KCR1 structure. Figure S4. Changes in PINs and auxin distribution in the kcr1‐2. Figure S5. Analysis of kcr1‐2 root and trichome phenotypes, KCR1 gene expression. Figure S6. Hypocotyl defects and abiotic stress sensitivity of kcr1‐2. [file TPJ-123-0-s004.docx]

**Supplementary Figure 1.** Apical phenotypes and allelic test to identify the *esm* mutation.

**A)** The *esm* mutation is recessive. Representative image of 7-day-old seedlings. **B)** *esm* is allelic to *kcr1*. 5-day-old seedlings. **C)** SEM images of apical patterning defects of dark-grown 5-day-old *esm* mutant seedlings. Plants were grouped into three phenotypic classes according to the most common dark-grown apical patterning defects. Penetrance of the phenotypes is depicted as percentages. **D)** Apical patterning defects of light-grown 5-day-old seedlings*.* Scale bars: A – 10mm, B, D – 5mm, C – 100 μm

**
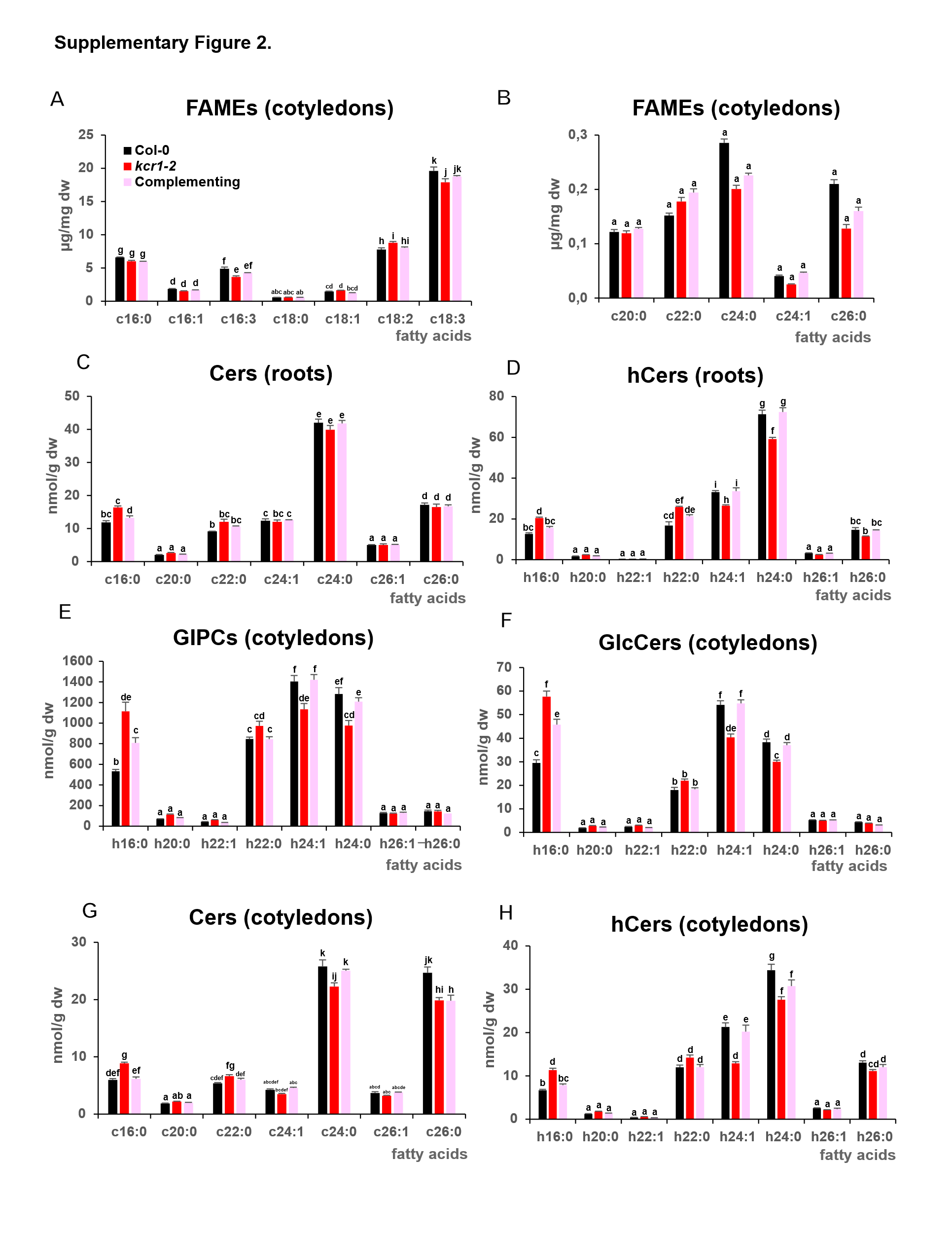
**

**Supplementary Figure 2**. VLCFA measurements in *kcr1-2* mutants

**A-B)** Fatty acyl methyl esters (FAMEs) profile in *kcr1-2* mutant shoots compared to wild type and complemented line (*pKCR1::KCR1-GFP* in *kcr1-2*). Total fatty acid levels in roots of *kcr1-2* mutant and complementing line compared to wild type (amounts expressed in µg/mg dry weight). Fatty acid values are the average of four samples ± SD. **C-H)** Sphingolipid profile in roots and shoots of *kcr1-2* mutant compared to wild type and complemented line. **C-D)** Ceramides (Cer) **(C)** and hydroxyceramides (hCer) **(D)** profiles in roots. **E-H)** glycosyl inositol phosphoceramides (GIPCs). **(E)**, glucosylceramides (GlcCers) **(F)**, Cer **(G)** and hCer **(H)** profiles in shoots. Color codes for genotypes are the same for all panels and shown in panel A. Sphingolipid amounts were shown according to their fatty acid length. For each hydroxylated (h) or non-hydroxylated (c) fatty acid, the values corresponded to the sum of the four LCBs isoforms. Sphingolipid values are the average of three samples ± SD. Statistical analyses were performed as described in Figure 2.

**Supplementary Figure 3.** Predictions of the KCR1 structure

**A)** AlphaFold3 model and map of electrostatic surface potential for KCR1^WT^. Black arrow highlights the site of the G184S *kcr1-2* missense mutation. **B)** AlphaFold3 model and map of electrostatic surface potential for KCR1^kcr1-2^. Black arrow highlights the site of the G184S *kcr1-2* missense mutation. **C)** AlphaFold3 model of the KCR1^kcr1-2^ mutant protein dimer (cyan) superimposed with that of wild-type KCR1 (grey). Black arrow highlights the G184S *kcr1-2* missense mutation. Coloring is arbitrary and does not reflect AF3 model confidence. **D)** AlphaFold3 model and a map of electrostatic surface potential for KCR1^WT^ dimer. **E)** AlphaFold3 model and a map of electrostatic surface potential for KCR1^kcr1-2^ dimer. **F)** AlphaFold3 prediction of a KCR1^WT^-KCS9-ECR-PAS2 heterotetramer in the presence of 50 myristates and 2 NADPH molecules. Uniprot IDs in the respective order: Q8L9C4, Q9SIX1, Q9M2U2, Q8VZB2. **G)** AlphaFold3 prediction of a KCR1^WT^-KCS9-ECR-PAS2 heterotetramer. Coloring highlights individual proteins. Coloring is arbitrary and does not reflect AF3 model confidence. **H)** Speculative interface between KCR1^WT^ and KCS9. **A, B, D, E, F)** AlphaFold3 structures show per-residue confidence score (pLDDT) distribution. **A, B, D, E)** Maps of electrostatic surface potential for AlphaFold3 protein models with units of k_B_T/ec ([Boltzmann constant x temperature] /the charge of an electron); coming from the solution of the Poisson-Boltzmann equation.

**
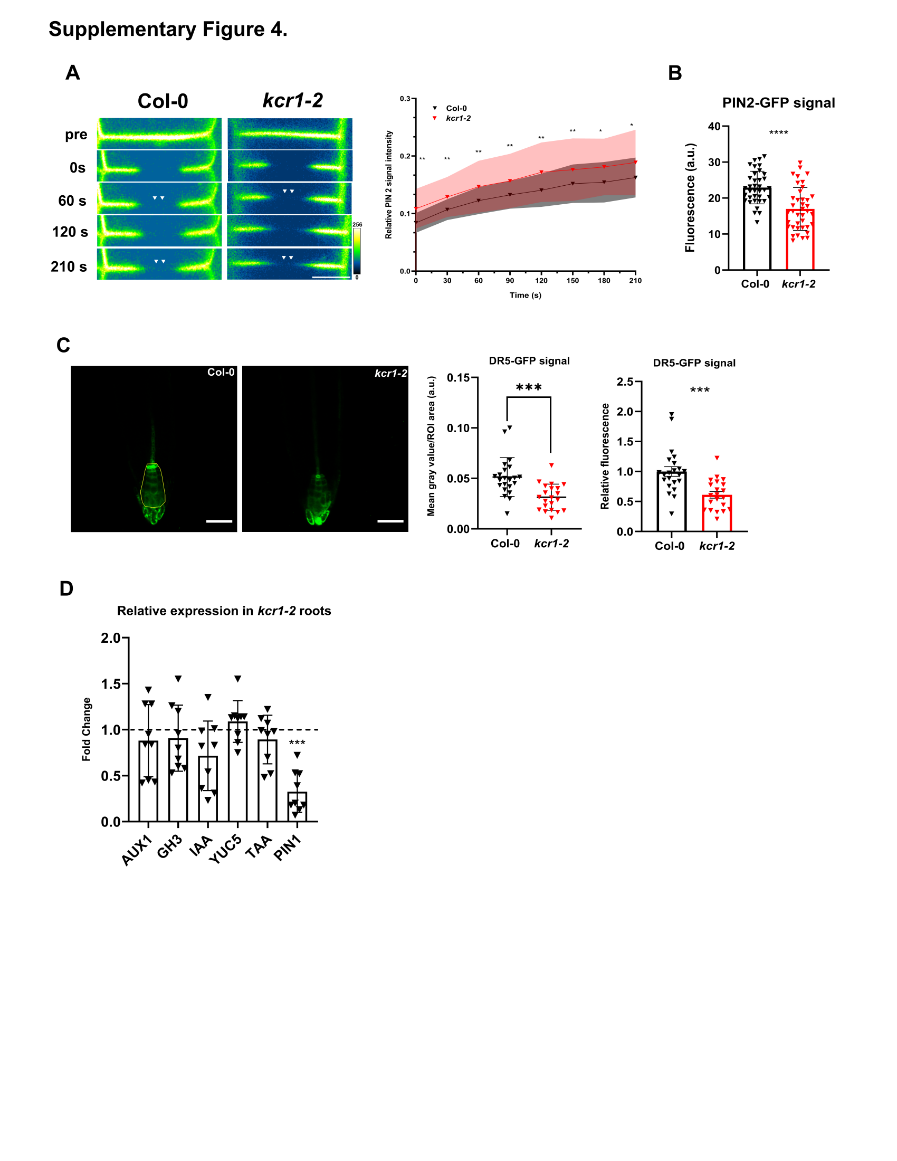
**

**Supplementary Figure 4.** Changes in PINs and auxin distribution in the *kcr1-2*.

**A)** Fluorescence Recovery After Photobleaching (FRAP) experiment on PIN2-GFP. Left panels: Representative images of PIN2-GFP before and after photobleaching with indicated time steps. Arrowheads indicate the bleached region of interest which was measured. Right panels: Quantification of the recovery of fluorescence signal. The images were taken every 30 seconds after bleaching. The data was shown as fluorescence signal normalized to the fluorescence intensity of the region of interest before bleaching. At least 5 cells in at least 5 different roots per genotype were imaged. Data is shown as mean ± SD. For statistical analyses, multiple Mann-Whitney tests were performed. (*, P<0.0332, **, P<0.0021).

**B)** Measurement of the PIN2-GFP signal on the membranes of root cells in indicated genotypes. n (Col-0) = 36; n (*kcr1-2*) = 39. Unpaired t-test with Welch’s correction was used (****, P<0.0001). **C)** Measurement of *DR5rev::GFP* signal in the QC and columella cells of the primary roots. The ROI for fluorescence signal measurements is indicated by yellow line. Measurements are shown as signal intensity per area (left graph) and normalized to signal in the wild type roots (right graph). For both approaches, an unpaired t-test with Welch’s correction was used (***, P=0.0003). **D)** Analysis of auxin-related genes’ transcript levels in *kcr1-2* seedling roots. The data was normalized to the complementing line which doesn’t significantly deviate from the wild type and is shown as dashed line. For statistical analyses, ordinary one-way ANOVA with Dunnett’s multiple comparisons test was used (***, P = 0.0006). Error bars show mean ± SD. Scale bars: A – 5 μm, C – 50 μm.

**
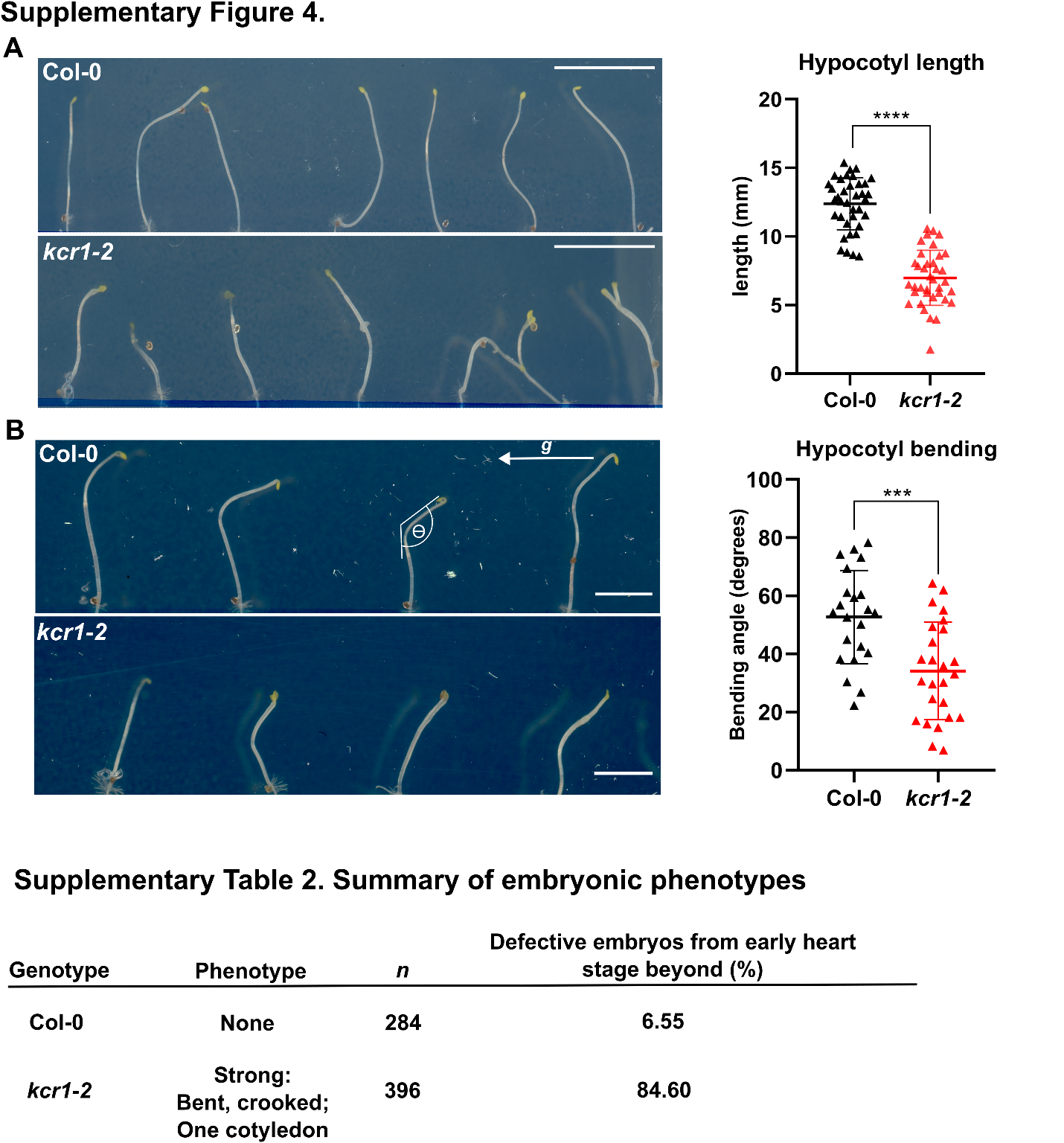
**

**Supplementary Table 2.** Summary of *kcr1-2* embryonic phenotypes.

Embryos were scored for deviation from the typical WT embryo patterning as percentage of all embryos observed. Representative images of the scored phenotype are visible in Figure 6A.

**
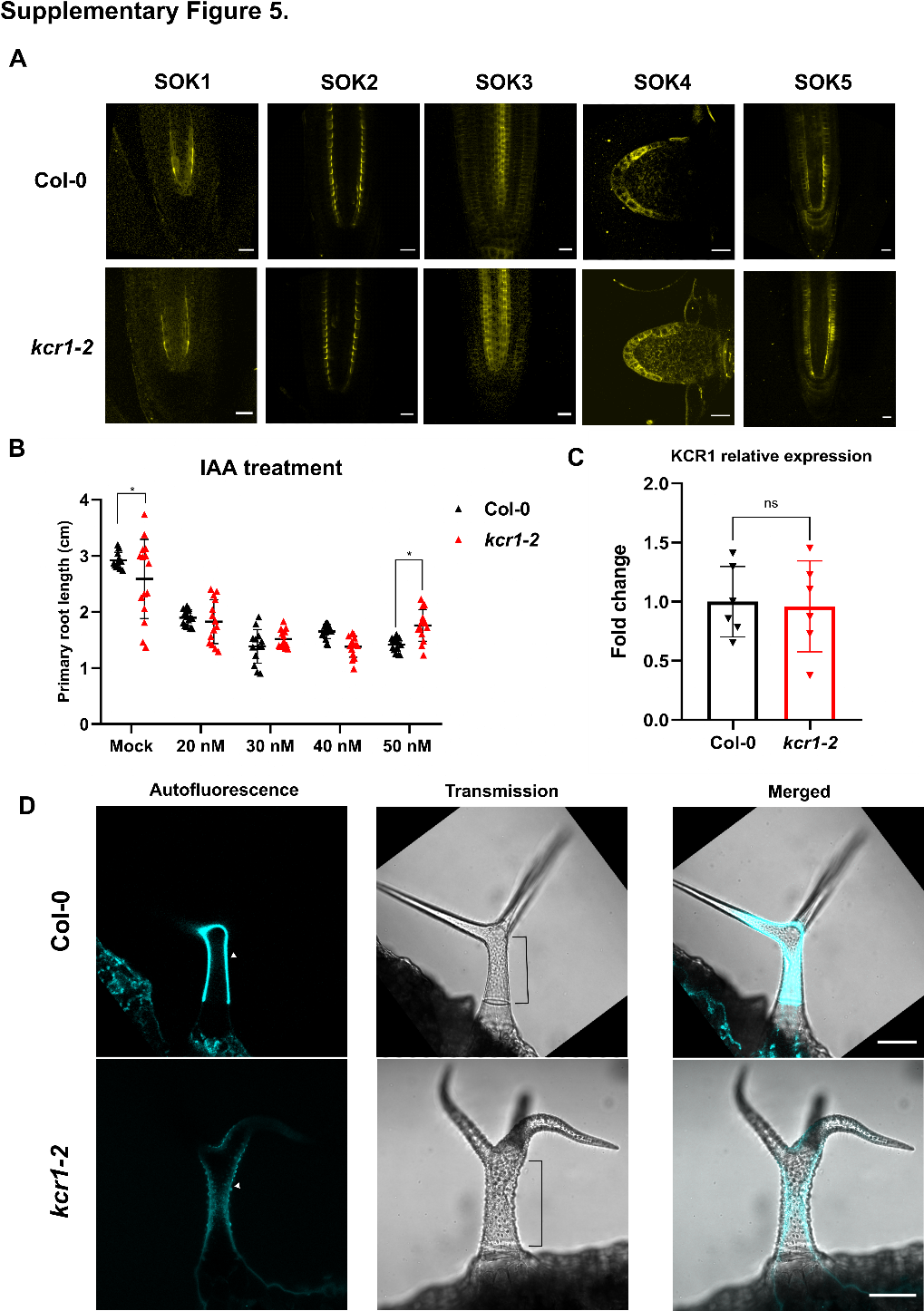
**

**Supplementary Figure 5.** Analysis of *kcr1-2* root and trichome phenotypes, *KCR1* gene expression.

**A)** Expression of SOSEKI (SOK) proteins in WT and *kcr1-2* primary or lateral roots. No significant polarity defect was observed in *kcr1-2* roots. **B)** Quantification of primary root length in response to increasing auxin (indole-3 acetic acid, IAA) concentration (Supplementary to Fig. 7B), focusing on the range of IAA concentrations between 20 nM and 50 nM. Two-way ANOVA with Sidak’s multiple comparison test. (ns, P > 0.05; **, P = 0.0012; ****, P value ≤ 0.0001). Mean ± SD. **C)** Analysis of *KCR1* transcript levels in whole seedlings by RT-qPCR. Unpaired two-sample t-test (ns). Mean ± SD. **D)** Trichome phenotype of *kcr1-2*. Left panels – cell wall autoflourescence measured in the DAPI channel. Note weaker autoflourescence signal in *kcr1-2* trichomes as compared to Col-0 (white arrowhead). Middle panels – brightfield images. Note increased number of papilli in the *kcr1-2* trichomes (within the black brackets). Right panels – merged image. Scale bars: A - 20 μm, D - 50 μm

**
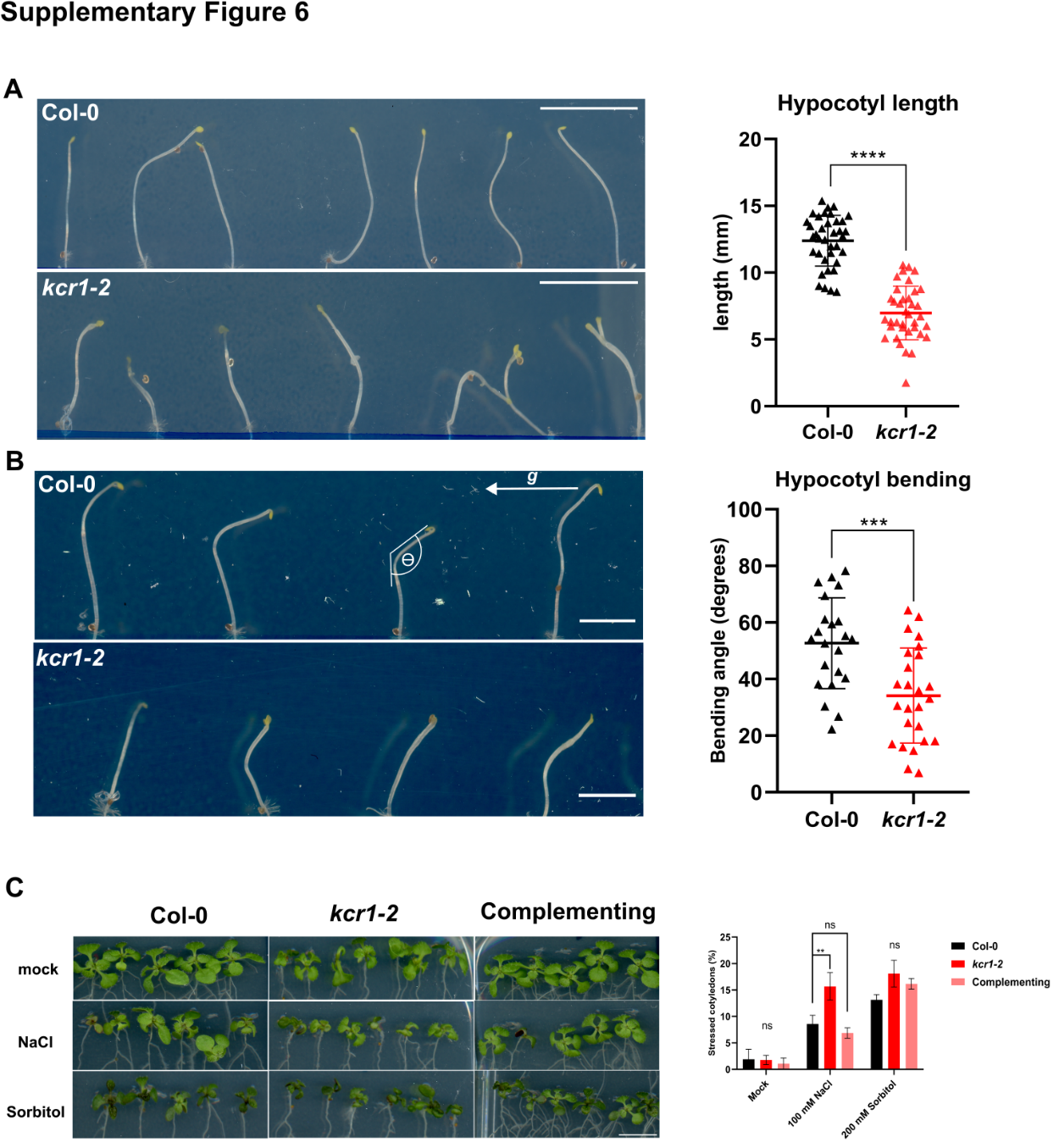
**

**Supplementary Figure 6.** Hypocotyl defects and abiotic stress sensitivity of *kcr1-2*

**A)** Hypocotyl length of 5-day-old dark-grown seedlings. Unpaired two-sample t-test (****, P ≤ 0.0001). **B)** Angle of hypocotyl bending upon gravistimulation. Theta represents the angle of bending that was measured. Arrow represents the gravity vector (g). Unpaired two-sample t-test (***, P = 0.0004). **C)** Abiotic stress resistance analysis. The seeds were germinated on normal MS plates and after 5 days were transferred to treatment plates containing mock treatment, 100 mM NaCl or 200 mM Sorbitol. After 7 days on the treatment plates, the seedlings were scored for cotyledons showing visible stress symptoms, such as full or partial chlorosis, anthocyanin accumulation (full or partial purple pigmentation) or any type of growth retardation. Left panels show the phenotypes and treatments. Right panels indicate the quantification. For statistical analyses, a two-way ANOVA with Dunnet’s multiple comparisons test (*kcr1-2/*complementing line vs Col-0 per treatment) was used (ns, P>0.1; **, P =0.0048). Scale bars: A, C – 10mm, B – 5mm.
