# Supplementary material for: Biosynthesis of very Long‐chain fatty acids is required for Arabidopsis auxin‐mediated embryonic and post‐embryonic development
Source: Plant J. 2025 Aug 9;123(3):e70396. doi: 10.1111/tpj.70396 (PMC12335294; doi:10.1111/tpj.70396)
Supplement: Supplementary file 4 — Table S3. Primers used in the study. [file TPJ-123-0-s001.pdf]

Supplementary Table 2. Primers used in the study

Primer name	Sequence	Purpose
KCR1_qPCR1_F	GATTGATGTTCAATGCCAGGTTCC	qPCR
KCR1_qPCR1_R	GATTGATGTTCAATGCCAGGTTCC	qPCR
pKCR1-FC-F	TGCCAACTTTGTATAGAAAAGTTGTAgtcctcttaaagctcgagct	Promoter cloning
pKCR1-FC-R	CGTTCAACTTTTTTGTACAAACTTGTtagagaagaaaggttgagac	Promoter cloning
KCR1_CDS_F	GGGGACAAGTTTGTACAAAAAAGCAGGCTTCATGGAGATCTGCACTTACTT	CDS cloning
KCR1_CDS_noStop_R	GGGGACCACTTTGTACAAGAAAGCTGGGTATTCTTTCTTCATGGAGTCTT	CDS cloning
Kcr1-1-GT-FP	CCCTCTGGTGATGCAACTAAG	*kcr1-1* genotyping
Kcr1-1-GT-RP	ATTGATGAAGGTGTGAAACGG	*kcr1-1* genotyping
Ds5-2a	TCCGTTCCGTTTTCGTTTTTTAC	*kcr1-1* genotyping
AUX1-rtF	TTCAGCTGCGCATCTAACCAA	qPCR

AUX1-rtR	TCTGTATTCGACGTAGAGAACAG	qPCR
AtIAA14-RTF	TCCCCGGAGGCACTGAA	qPCR
AtIAA14-RTR	AAGCCTCTCTTGTTCCCAACAC	qPCR
yuc5-rtf	ACGCGTGGAAAGGGAAATCG	qPCR
yuc5-rtr	CGGTGACCCACTCGTGTTCT	qPCR
SAUR19 F	CTTCAAGAGCTTCATAATAATTCAAACTT	qPCR
SAUR19 R	GAAGGAAAAAATGTTGGATCATCTT	qPCR
GH3.5_F	AGGCCAGTGTTGTTGTCTTTG	qPCR
GH3.5_R	TGGTCTTGAGCATAGATTCCG	qPCR
TAA1_qF	GATGAAGAATCGGTGGGAGA	qPCR
TAA1_qR	CGGACATGCTTCTTGTCAGA	qPCR
PIN1_qPCR_F	ACAAAACGACGCAGGCTAAG	qPCR
PIN1_qPCR_R	AGCTGGCATTTCAATGTTCC	qPCR
PP2A FW RT-qPCR	TAACGTGGCCAAAATGATGC	qPCR
PP2A RE RT-qPCR	GTTCTCCACAACCGCTTGGT	qPCR
